# An Untargeted Metabolomics Approach for Correlating Pulse Crop Seed Coat Polyphenol Profiles with Antioxidant Capacity and Iron Chelation Ability

**DOI:** 10.3390/molecules26133833

**Published:** 2021-06-23

**Authors:** Fatma M. Elessawy, Albert Vandenberg, Anas El-Aneed, Randy W. Purves

**Affiliations:** 1 College of Pharmacy and Nutrition, University of Saskatchewan, Saskatoon, SK S7N 5E5, Canada; fatmaelzahraa.elessawy@usask.ca (F.M.E.); anas.el-aneed@usask.ca (A.E.-A.); 2 Department of Plant Sciences, University of Saskatchewan, Saskatoon, SK S7N 5A8, Canada; bert.vandenberg@usask.ca; 3 Centre for Veterinary Drug Residues, Canadian Food Inspection Agency, Saskatoon, SK S7N 2R3, Canada

**Keywords:** antioxidant activity, iron binding, pulse crops, anthocyanins, proanthocyanidins, flavan-3-ols, untargeted metabolomics

## Abstract

Pulse crop seed coats are a sustainable source of antioxidant polyphenols, but are typically treated as low-value products, partly because some polyphenols reduce iron bioavailability in humans. This study correlates antioxidant/iron chelation capabilities of diverse seed coat types from five major pulse crops (common bean, lentil, pea, chickpea and faba bean) with polyphenol composition using mass spectrometry. Untargeted metabolomics was used to identify key differences and a hierarchical analysis revealed that common beans had the most diverse polyphenol profiles among these pulse crops. The highest antioxidant capacities were found in seed coats of black bean and all tannin lentils, followed by maple pea, however, tannin lentils showed much lower iron chelation among these seed coats. Thus, tannin lentils are more desirable sources as natural antioxidants in food applications, whereas black bean and maple pea are more suitable sources for industrial applications. Regardless of pulse crop, proanthocyanidins were primary contributors to antioxidant capacity, and to a lesser extent, anthocyanins and flavan-3-ols, whereas glycosylated flavonols contributed minimally. Higher iron chelation was primarily attributed to proanthocyanidin composition, and also myricetin 3-*O*-glucoside in black bean. Seed coats having proanthocyanidins that are primarily prodelphinidins show higher iron chelation compared with those containing procyanidins and/or propelargonidins.

## 1. Introduction

Pulse crops are harvested for dry seeds used in human diet as they are excellent sources of protein, carbohydrates and micronutrients, such as minerals, vitamins and bioactives [1]. Among these bioactive components, polyphenols, a group of secondary metabolites, are typically concentrated in seed coats [2]. Structurally, polyphenols are classified as flavonoids characterized by a diphenylpropane (C_6_-C_3_-C_6_) basic skeleton, and non-flavonoids [3]. Flavonoids are the most abundant class in pulse crops and are categorized into monomers or complex polymers (Figure S1). Flavonols and anthocyanins are typically the most abundant flavonoid monomers, whereas procyanidins and prodelphinidins are the most common types of polymeric flavonoids, known as proanthocyanidins [4]. Functionally, polyphenols not only protect seeds against UV radiation, pathogens and environmental stresses, but they can also provide humans with health benefits mainly due to their free radical scavenging ability via electron transfer to unstable radicals [5,6]. These radicals are normally produced in the body during metabolism and are required for certain functions, such as destruction of pathogenic microbes and regulation of intracellular signaling cascades [7]. However, when generated in excess, they exert harmful effects due to oxidative stress that can induce cellular damage for biomolecules, including DNA, proteins and lipids. High levels of free radicals are contributors to the progression of aging and several diseases, such as atherosclerosis, type 2 diabetes and cancer [8,9].

Some polyphenols form polyphenol-iron complexes that reduce iron bioavailability and lead to iron deficiency and anemia, which is associated with poor health, serious functional impairments in human and increased risk of mortality [10]. The prevalence of iron deficiency-related anemia is significantly higher in the developing countries where protein nutrition predominantly depends on pulse crops [11]. Hart et al., reported that myricetin, quercetin and their 3-*O*-glucosides prevented iron absorption through complex formation with iron, whereas catechin, epicatechin, kaempferol and 3,4-dihydroxybenzoic acid significantly promoted iron uptake in an in vitro model using *Caco-2* cells [12]. The exact mechanism behind either inhibition or promotion of iron uptake is still unclear.

Despite containing iron chelating polyphenols, pulse crop seed coats have been reported as sustainable sources of natural polyphenolic antioxidants [13,14,15]. For example, Duenas et al., observed that the antioxidant capacity of the seed coat extracts was higher than the cotyledon extracts in both lentil and pea [16]. Flavones, flavonols and proanthocyanidins had the greatest contribution to the antioxidant capacity of the lentil seed coat extract, whereas catechin containing compounds were the main contributors to the antioxidant activity of the lentil cotyledon extract [16]. Despite their antioxidant activity, seed coats are often removed before processing, cooking or consumption and used as animal feed [17]. Recently, food scientists have begun exploring more effective uses of these seed coats by adding them to processed foods. For example, pea hull fiber was added to whole wheat bread [18] and cookies [19], whereas roasted hulls from navy and pinto beans, and black-eyed peas were included in muffins to enhance texture and moisture content [20]. Beside food applications, seed coat polyphenols, as antioxidants, can be used as green preservatives in industrial smart food packaging [21]. Water extract of lentil seed coats was used to control lipid peroxidation in chicken bologna without changing texture or sensory properties [22]. Additionally, polyphenols have recently been incorporated as active ingredients in cosmetic formulations and skin care products, as polyphenols provide photo-protection and prevent premature aging [23]. Accordingly, valorization and recycling of agri-food byproducts, such as pulse seed coats, could be achieved through potential use of pulse polyphenols in industrial, medicinal and cosmetic applications [24,25].

For effective use and valuation of pulse crop seed coats, investigation of both antioxidant and iron chelation capabilities of their polyphenols is crucial. Polyphenol classes in seed coats will have different antioxidant and iron chelation capabilities, but currently limited information is available regarding these correlations. Previous work with a targeted LC-MS method [26] is used to help guide this study, but to properly bridge this gap, the major polyphenol classes responsible for these capabilities will be investigated using an untargeted metabolomics analysis based on liquid chromatography-high resolution mass spectrometry (LC-HRMS). With the aid of multivariate statistical analysis, the aim is to use untargeted metabolomics to provide insight into the metabolites (mainly polyphenols) responsible for differentiating antioxidant capacity among cultivars. Untargeted metabolomic approaches have been applied previously to investigate metabolite diversity, including among apple, pear and peach cultivars having different antioxidant activity [27,28]. In this study, identification of the major polyphenols by untargeted methods will enable the relationship between polyphenol composition in seed coats of the five major pulse crops (chickpea, faba bean, lentil, pea, and common bean) to be compared with their chelation ability and antioxidant capacity using multiple assays. These assays measure different mechanisms of antioxidant capacity and create unique antioxidant profiles for each seed coat sample. Due to the vast complexity of structures of the proanthocyanidins, an additional assay was used to measure total proanthocyanidin content to better assess the contribution of proanthocyanidins to the measured antioxidant capacity and iron chelation. The main objective of this study was to investigate the antioxidant potential and iron chelation of the seed coat extracts and correlate these results with their polyphenol profiles using LC-HRMS based untargeted metabolomics. This study will provide important information for plant breeders and help with the valorization of seed coats, which are currently often discarded as low value waste.

## 2. Results and Discussion

Extracts of four different seed coat genotypes of chickpea, faba bean, lentil, pea and common bean were used to investigate the relationship between pulse crop seed coat polyphenols and in vitro antioxidant capacity/iron chelation ability. In addition to four different antioxidant assays, an iron chelation and a proanthocyanidin assay were also employed. Correlating these assay results with the polyphenol profiles, involved the use of an LC-HRMS based untargeted metabolomics approach.

### 2.1. Assay Results and Evaluation of the Antioxidant Capacity Assays

**Figure 1:**
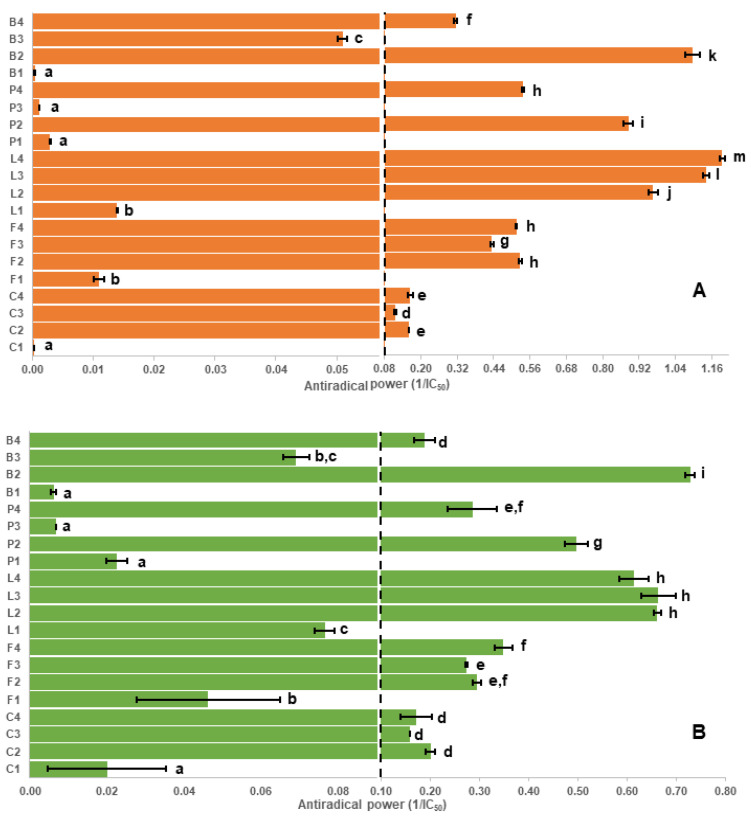
Antioxidant capacity of different genotypes of pulse seed coats measured by DPPH (**A**) and TBARS (**B**) assays. Letters on y-axis refer to different pulse crops; C: chickpea, F: faba bean, L: lentil, P: pea and B: common bean, whereas numbers refer to different seed coat colors. For example, L2 refers to black seed coats of Indianhead lentil (see Table 3, Section 3.1 for code). ARP: antiradical power; ARP is the reciprocal of IC_50_. Bars with different letters for each assay are significantly different (*p* < 0.05).

**Figure 2:**
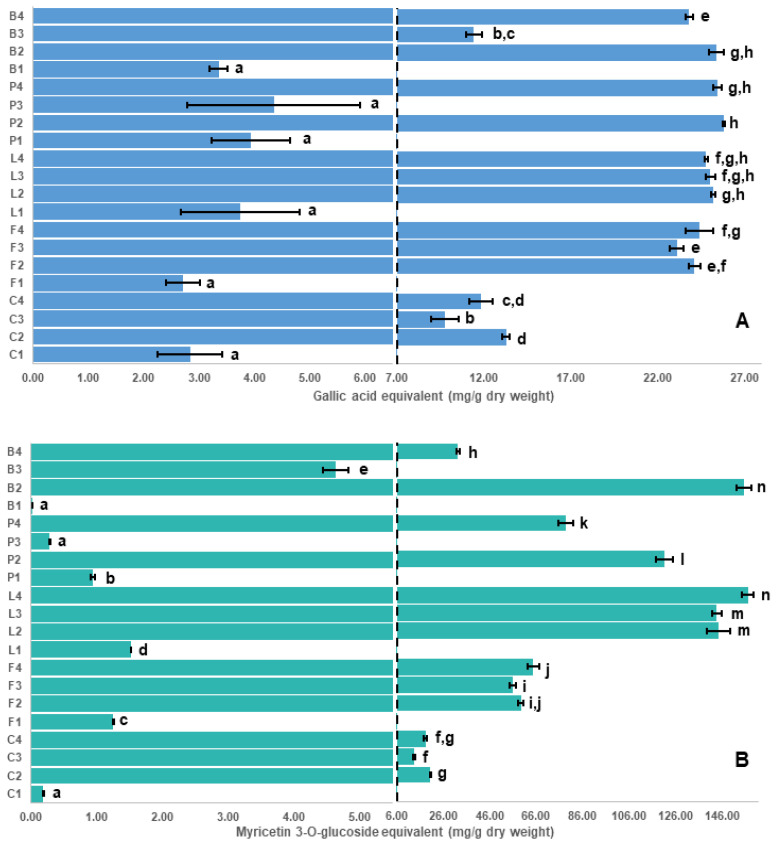
Antioxidant capacity of different genotypes of pulse seed coats measured F-C (**A**) and FRAP (**B**) assays. Letters on y-axis refer to different pulse crops; C: chickpea, F: faba bean, L: lentil, P: pea and B: common bean, whereas numbers refer to different seed coat colors. For example, L2 refers to black seed coats of Indianhead lentil (see Table 3 for codes). F-C: Folin–Ciocalteu assay; FRAP: Ferric reducing antioxidant power. Bars with different letters for each assay are significantly different (*p* < 0.05).

**Figure 3:**
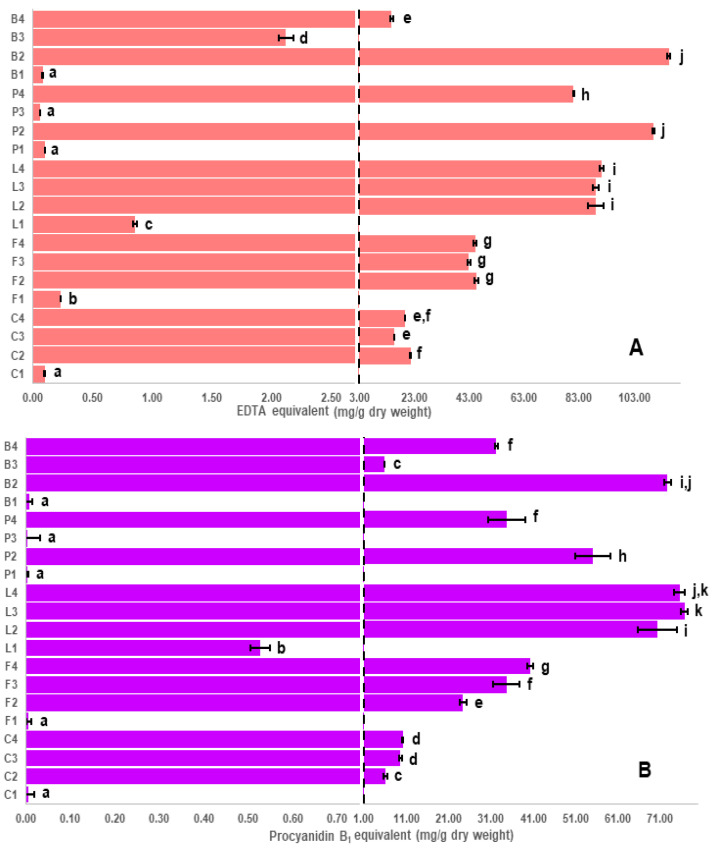
Iron chelation ability (**A**) and proanthocyanidin content (**B**) of different genotypes of pulse seed coats using Ferrozine and DMAC assays, respectively. Letters on y-axis refer to different pulse crops; C: chickpea, F: faba bean, L: lentil, P: pea and B: common bean, whereas numbers refer to different seed coat colors. For example, L2 refers to black seed coats of Indianhead lentil (see Table 3 for code). Bars with different letters for each assay are significantly different (*p* < 0.05).

Results for the various assays used in this study are shown in Figure 1, Figure 2 and Figure 3. Despite some limitations [29,30], in vitro antioxidant assays are commonly used to measure the antioxidant capacity and enable the investigation of a broad scope of possible antioxidant mechanisms of phytochemicals, such as polyphenols. The four antioxidant assays used in this study, DPPH radical (Figure 1A), thiobarbituric acid reactive substances (TBARS, Figure 1B), Folin–Ciocalteu (F-C, Figure 2A) and ferric reducing antioxidant power (FRAP, Figure 2B) show similar patterns for the seed coat extracts (Table S2). In addition to the antioxidant assays, a ferrozine assay [31] was used to measure the iron chelation ability (Figure 3A, Table S3) and a 4-dimethylaminocinnamaldehyde (DMAC) assay [32] was used to estimate the total amount of proanthocyanidins (a diverse class of polyphenols containing numerous isomeric oligomers, Figure 3B, Table S3).

Table 1 shows a high correlation (>0.97) among three of the antioxidant capacity assays (DPPH, TBARS, FRAP); however, correlations between these three assays and the F-C assay are much lower (<0.87). The F-C assay appears to overestimate the antioxidant capacity of all low tannin seed coat extracts compared with DPPH, FRAP and TBARS assays. The interaction between the F-C reagent and some reducing agents in the sample (e.g., amino acids, aromatic amines and citric acid), can yield a higher color intensity that interferes with the analysis [33]. Additionally, the F-C assay was incapable of distinguishing among the highest antioxidant capacities (Figure 2A). This observation could be explained by the oxidation of most phenolic compounds, found as phenolate anions at the assay pH (~10), by the F-C reagent causing an overestimated value of the antioxidant capacity [34]. Thus, the F-C assay was considered as the least reliable antioxidant assay in this study and its use is not recommended.

### 2.2. Correlation of Antioxidant Capacity to Polyphenol Classes

**Figure 4:**
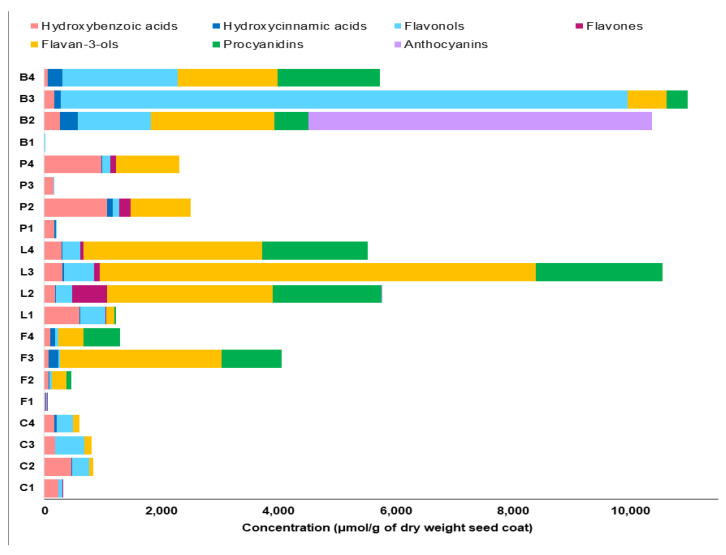
Cumulative concentrations of major polyphenol subclasses in seed coat extracts in µmol/g dry weight using the *targeted method*. This figure was derived from Elessawy et al. [26] and the concentrations of individual polyphenols are given in Tables S4–S8. Letters on y-axis refer to different pulse crops; C: chickpea, F: faba bean, L: lentil, P: pea and B: common bean, whereas numbers refer to different seed coat colors. For example, L2 refers to black seed coats of Indianhead lentil (see Table 3 for code).

Initially, the antioxidant potential measured by the assays was correlated to the concentrations of polyphenol classes measured using our recently established targeted LC-SRM method [26], where concentrations of 8 major polyphenol classes in the pulse crop seed coats are shown in Figure 4 and Tables S4–S8. In Table 1, flavan-3-ols and procyanidins showed the highest correlation with the measured antioxidant capacity. In general, low antioxidant capacity values (statistically significant at *p* < 0.05) were found in the low tannin species, which express genes that lower specific polyphenol concentrations, especially flavan-3-ols and proanthocyanidins. These genotypes include kabuli chickpea (C1), white-flowered faba bean (F1), white common bean (B1), white pea (P1), and green pea (P3) seed coat extracts. Conversely, high antioxidant capacity was found in seed coat extracts having high amounts of polyphenols, such as black (L2), green (L3) and brown (L4) lentil; and black common bean (B2). These findings are consistent with previous studies that reported that light-colored legume seeds showed low antioxidant activity compared to dark-colored seeds [35,36,37]. However, there were some notable exceptions, especially in the yellow common bean (B3) seed coat extract. This is because, in addition to the amount of polyphenols present, the polyphenol class (Figure S1) is also critically important, as will be described in more detail below.

The seed coat extracts of black common bean (B2), and black (L2), green (L3) and brown (L4) lentil were found to have the highest antioxidant capacity (statistically significant at *p* < 0.05), which is most likely attributed to their high content of flavan-3-ols and procyanidins that protect against lipid peroxidation. The importance of these classes is supported by Verstraeten et al. who found that flavan-3-ols and procyanidins form hydrogen bonds with the polar head groups of the liposome phospholipids that accumulate outside and inside the liposome membranes protecting against induced oxidative damage [38]. Although both flavan-3-ols and procyanidins are effective antioxidants, results from Hagerman et al. suggested that condensed tannins (e.g., proanthocyanidins) were significantly more effective as peroxyl radical quenchers than flavan-3-ols [39].

In addition to flavan-3-ols and procyanidins, black common bean (B2) seed coat extract was unique among all seed coats examined as it contains a large amount of anthocyanins (Figure 4) that are also major contributors to the antioxidant capacity. Anthocyanin stability is pH dependent showing high stability at acidic pH (in the form of flavylium cation), whereas their structure is expected to change at neutral pH. The reaction medium in TBARS and FRAP assays is acidic (where anthocyanins are stable), however, even in neutral media (in DPPH assay), anthocyanins were reported to show antioxidant activity [40]. Tsuda et al. reported that anthocyanins isolated from black seed coats of common bean, especially delphinidin and delphinidin 3-*O*-glucoside, showed high antioxidant activity by reducing the formation of malondialdehyde in a liposomal system and enhancing the superoxide anion (O^2●–^) scavenging activity at neutral pH [40]. The lower correlation for anthocyanins reported in Table 1 is likely because of the small sample size (only one seed coat sample had a high anthocyanin level) involved in calculating this correlation [41]. Finally, Table 1 also suggests that flavonols, have little effect on antioxidant capacity. For example, the extract of the yellow common bean (B3) seed coat has the largest amount of flavonols (mainly kaempferol 3-*O*-glucoside) among the 20 genotypes in this study (Table S4), but the lowest antioxidant capacity among the tannin containing genotypes.

Although a high correlation has been previously observed between the antioxidant capacity and procyanidin content [42,43], procyanidins are only a subclass of polymeric polyphenols (proanthocyanidins) that also includes prodelphinidins and propelargonidins. An accurate estimation of the total amount of proanthocyanidins by LC-MS is extremely difficult because there are hundreds of these compounds, which have numerous structural isomers. Since the vast majority of them are not commercially available as standards, the DMAC assay was used to estimate proanthocyanidin levels (Figure 3B, Table S3). Correlations to this assay shown in Table 1 suggest that although there is a correlation between flavan-3-ol and procyanidin content with antioxidant capacity, it is relatively weak compared with the correlation between proanthocyanidin level, as measured by the DMAC assay, and antioxidant capacity. This finding suggests that other proanthocyanidins, not detected in the targeted method, are expected to play an important role in the measured antioxidant capacity. Thus, although the targeted method is useful in guiding the analysis, it has limitations, and therefore to obtain a thorough understanding of the key polyphenols responsible for high antioxidant capacity, an untargeted LC-MS method was employed.

### 2.3. Untargeted Analysis of the Seed Coat Samples

**Figure 5:**
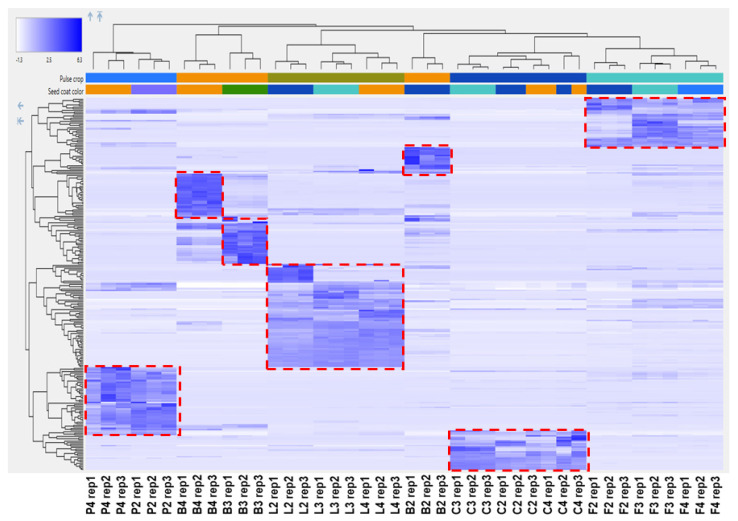
Heat map of hierarchal clustering analysis (HCA) showing separate clusters for seed coat samples.

To explore the polyphenolic variations among the pulse seed coats, an untargeted approach was applied. Principal component analysis (PCA) plots (PC1 versus PC2) of the individual pulse crops all showed a clear separation between the detected metabolites from the low tannin and the tannin-containing genotypes (Figure S3). As the tannin containing genotypes are known to have the highest antioxidant capacities [44] and the low tannin genotypes do not contribute meaningfully (as was observed in Figure 1 and Figure 2), a subsequent untargeted analysis of all pulse crops was restricted to only seed coats from tannin containing genotypes. The analysis initially yielded thousands of compounds (the amount is highly dependent on the area cut-off used in the analysis). To reduce this to a more workable number and focus only on the more intense polyphenols, additional filters were used (Section 3.8) resulting in 235 compounds. A hierarchical cluster analysis (HCA) of these 235 compounds shows a heat map of the major metabolite distribution among seed coats where the color intensity of each rectangle represents the relative amount (by area) of a specific metabolite in a specific sample. The HCA heat map shows seven distinct clusters (Figure 5), as biological replicates group together. Only in common bean, do the seed coats separately cluster, implying major differences among the major compounds in each colored common bean seed coat. Conversely, colored lentil, chickpea, faba bean and pea seed coats were found to show primarily one main cluster for each crop, although some minor differences are noted. The differences suggest the polyphenol profiles are less diverse within these crops compared with common bean. After checking the metabolites in each cluster, they were identified, with varying levels of confidence, by comparing their MS2 spectra with online and in-house databases (Table 2). The identification levels in the table follow Sumner et al., with the addition of level 2/3 indicating isomeric compounds, such as those for the proanthocyanidin oligomers [45].

When checking identifications of compounds in the hierarchical plots using Compound Discoverer 3.1, some redundancy was observed. Although the software groups isotopes and adducts, for larger molecular weight species, and especially for the proanthocyanidin oligomers, an incorrect isotopic peak was occasionally selected as the monoisotopic peak (primarily caused by overlapping oligomer isotopes) and two entries were reported. These redundant assignments were removed from the table. Similarly, some adducts, particularly dimers and multimers (e.g., [M + 2H]^3+^) that were reported at the same retention time as the monomer (and showed fragmentation back to the monomer) were also removed from the table. However, one type of adduct was not removed as it was the dominant form of the compound. Anthocyanins were mainly detected as water adducts in Table 2; the preferential presence of these intense anthocyaninin water adduct ions observed in negative mode has been described previously [46]. Thus, although the software provided an excellent starting point, these results highlight the importance of carefully examining each entry.

Table 2 also shows many polyphenols detected with the untargeted method that were not quantified by our targeted method (all 19 compounds in bold are polyphenols in our targeted method). Many of the polyphenols in the table do not have commercially available standards necessary for absolute quantification, however, an estimate of the levels of the major compounds is important in establishing the contribution of the polyphenol classes to various assays. Consequently, the amounts of the polyphenols in Table 2 were estimated using related polyphenols (e.g., kaempferol 3-*O*-rutinoside was used for all kaempferol compounds, procyanidin B1 was used for all proanthocyanins, etc.) and the estimated amounts are shown in Supplementary Tables S9–S13. Note that we emphasize the amounts in these Tables S9–S13 are *estimates* as absolute quantification can only be achieved using authentic standards.

The hierarchical plot (Figure 5) and Supplementary Table S9 both show that the polyphenols most abundant in yellow common bean (Table 2) include glycosylated flavonols, and to a lesser extent, phenolic acids, flavan-3-ols (epi-afzelechin and afzelechin) and propelargonidins containing (epi)afzelechin (note this notation indicates that either epi-afzelechin or afzelechin can be present), which are a type of proanthocyanidins. Glycosylated flavonols were found to be less potent antioxidants than their corresponding aglycones [47]. For example, it was found that the glycosylation of flavonols, such as quercetin and kaempferol, significantly reduced the peroxynitrite scavenging activity [48]. Accordingly, although the yellow common bean seed coat extract (B3) has the highest amount of kaempferol 3-O-glucoside (Table S4) among all the seed coat extracts, the contribution of kaempferol 3-*O*-glucoside to the measured antioxidant activity of B3 was minimal. This finding is also supported by Cai et al. who reported that the substitution of 3-hydroxyl group by a sugar moiety in flavonoids, such as kaempferol-3-*O*-glucoside, diminished their antioxidant activity compared to their aglycones [49]. Additionally, it was found that kaempferol mono-, di- and triglycosides were significantly less potent as lipid peroxidation inhibitors compared to the kaempferol aglycone [50].

**Figure 6:**
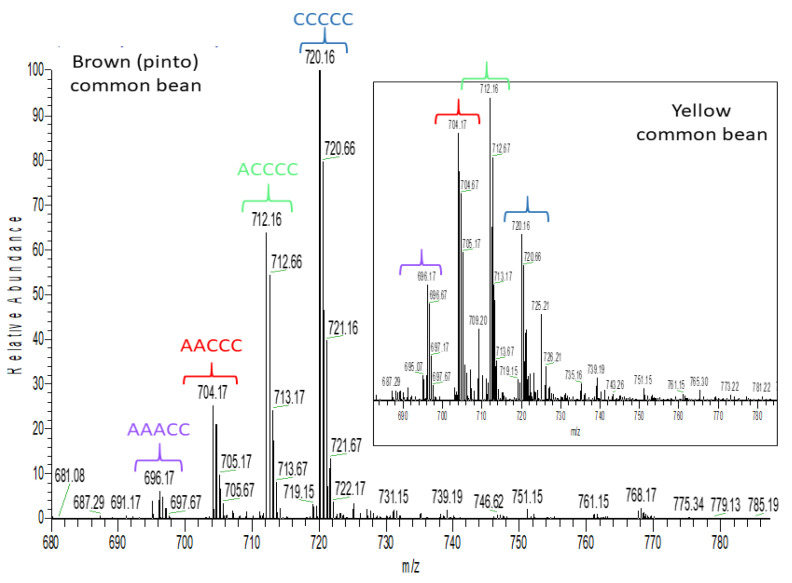
High resolution mass spectrometric (HRMS) full scan showing a procyanidin pentamer and different propelargonidin pentamers detected in yellow and brown common bean seed coats. Brackets with the same color have the same pentamer structure. C: (epi)catechin and A: (epi)afzelechin.

**Figure 7:**
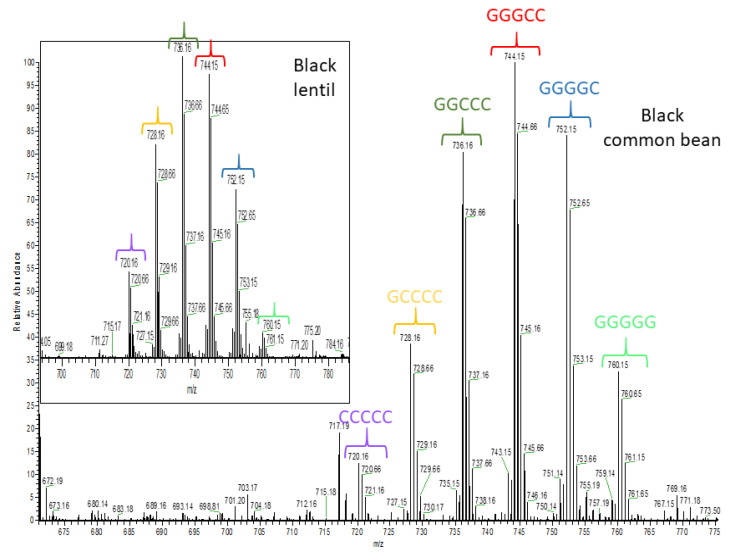
High resolution mass spectrometric (HRMS) full scan showing a procyanidin pentamer and different prodelphinidin pentamers detected in black common bean and lentil seed coats. Brackets with the same color have the same pentamer structure. C: (epi)catechin and G: (epi)gallocatechin.

The two other common bean clusters show very different polyphenol profiles. The brown common bean cluster (Table 2) shows exclusively procyanidins and propelargonidins. Figure 6 shows mass spectra for the doubly charged pentamers of yellow and brown bean, which were the only seed coats in this study where abundant propelargonidins were observed. Note that the distribution for the pentamer is very similar for other proanthocyanidin oligomers in these seed coats (not shown). Conversely, the black common bean cluster consists of primarily prodelphinidins, an anthocyanin water adduct, and also some glycosylated flavonols. Figure 7 shows how the distribution of the doubly charged pentamers is different in black bean compared with yellow or brown bean (Figure 6). Note that only a few prodelphinidins are indicated in Table 2, because several other prodelphinidins are shared with other pulse crops. Table S9 shows estimated amounts for the major proanthocyanidins found in bean.

The data suggests that oligomeric proanthocyanidins are major contributors to the antioxidant capacity and Figure 3B (and Table S9) shows that black and brown common bean seed coats have the greatest number of these oligomers, whereas yellow beans have the fewest. The antioxidant capacity of monomeric and polymeric flavonoids (proanthocyanidins) depends on factors such as structure, number of phenolic hydroxyl groups and degree of polymerization [51]. For example, prodelphinidin dimers, purified from pomegranate peel, showed more potent antioxidant capacity than gallocatechin monomers through inhibiting lipid peroxidation of liposomes [52]. The combination of 5- and 7-hydroxyl groups in the A-ring of proanthocyanidins (see Figure S2X), as a 2,4-substituted resorcinol substructure significantly enhances the antioxidant capacity [53]. Furthermore, the presence of the *O*-dihydroxy structure in the B-ring, and 2,3-double bond in conjugation with a 4-oxo function in ring C, as illustrated in Figure S2X, are also criteria controlling antioxidant capacity [54]. In this study, the antioxidant capacity of black common bean seed coats compared with brown common bean seed coats is higher not only because of the higher concentration of proanthocyanidins (as estimated by the DMAC assay, Figure 3B), but also likely because of the presence of anthocyanins, which are also strong contributors to antioxidant capacity [40].

As the antioxidant capacities of colored lentil seed coat genotypes were similar, it was expected that common compounds among these genotypes likely contributed the most to their antioxidant capacity. The heat map (Figure 5) shows a shared cluster (Table 2) that includes many procyanidins and prodelphinidins and the estimated amounts of the lentil polyphenols identified in Table 2 are given in Table S10. Figure 7 shows how the distribution of the doubly charged proanthocyanidin pentamers in black lentil is similar to black bean. Note that the distributions of the doubly charged proanthocyanidin pentamers of green and brown lentil (not shown) were very similar to black lentil. There are some compounds specific to each color, but unlike the common beans, outside of the one anthocyanin and a couple of extra prodelphinidins found in black lentil, there are no major differences among the polyphenols that make up the classes as each color contains several proanthocyanidins and flavonol glycosides. The lack of major differences across polyphenol classes is consistent with the similar antioxidant activity that was observed.

**Figure 8:**
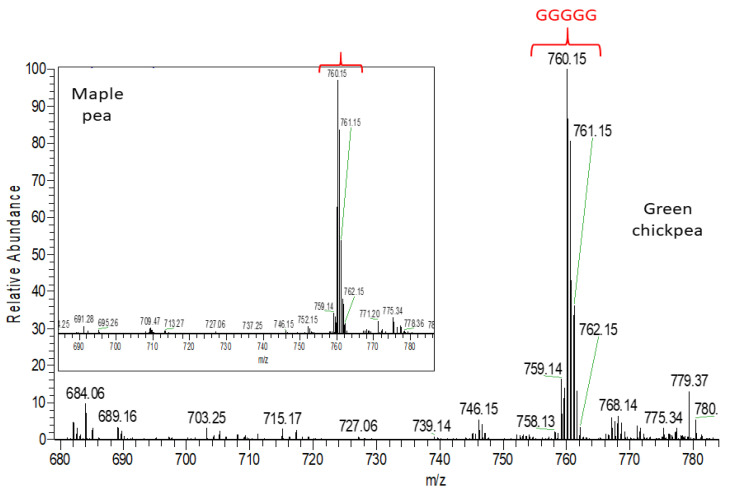
High resolution mass spectrometric (HRMS) full scan showing a prodelphinidin pentamer detected in chickpea and pea seed coats. Brackets with the same color have the same pentamer structure. G: (epi)gallocatechin.

In addition to lentil and common bean seed coats in the heat map, maple (P2) and dun (P4) pea, showed a common cluster (Figure 5) mostly including amino acid derivatives [55] and prodelphinidins (estimated polyphenol amounts for pea are given in Table S11). Figure 8 shows the distribution of the doubly charged proanthocyanidin pentamers in maple pea (brown pea was similar). Unlike Figure 7 in which the prodelphinidins contained both (epi)gallocatechin and (epi)catechin (labelled “G-C”-prodelphinidins” in the Supplementary Tables), Figure 8 shows almost exclusively (epi)gallocatechin prodelphinidin pentamers (labelled “G”-prodelphinidins in the Supplementary Tables). The prodelphinidins contribute to the antioxidant potential [56,57] and the reason for the differences in antioxidant potential in P2 and P4 can be mostly attributed to their differences in proanthocyanidin concentration as was shown in Figure 3B.

Colored chickpea seed coat genotypes showed a shared cluster (Table 2) including mostly phenolic acids and glycosylated flavonols, but also some prodelphinidins (estimated polyphenol amounts for chickpea are given in Table S12). Figure 8 shows the distribution of doubly charged proanthocyanidin pentamers in green chickpea (other tannin chickpea were similar). Colored faba bean seed coats also showed a shared cluster (Table 2) mainly containing phenolic acids and prodelphinidins (estimates of polyphenol amounts in faba bean are given in Table S13). Figure S4 shows the distribution of doubly charged proanthocyanidin pentamers in black faba bean (again other tannin faba beans were similar). These shared clusters within these pulse crops indicate similar polyphenolic profiles among colored seed coats in both chickpea and faba bean, resulting in insignificant differences in their antioxidant capacities.

### 2.4. Correlation of Iron Chelation to Polyphenol Classes

Iron chelation ability, defined as the capability of a polyphenol to chelate iron ions, (Figure 3A, Table S3) is dependent on the chemical structure as well as the number and the position of hydroxyl groups. In this study, the highest iron chelators were as follows: black (B2) common bean > maple (P2) pea > black (L2) ~ green (L3) ~ brown (L4) lentil. The correlation between iron chelation ability and the DMAC proanthocyanidin content (Table 1) suggests that iron chelation ability is highly dependent on the proanthocyanidin concentration in seed coat extracts. However, the order of proanthocyanidin content among the seed coats with the highest iron chelation ability was somewhat different, green (L3) ~ brown (L4) > black (L2) lentil ~ black (B2) common bean > maple (P2) pea. These differences in order can be largely explained by the composition of the proanthocyanidins. As (epi)gallocatechins have 3,4,5-tri-hydroxyl groups in ring B, (an additional hydroxyl group compared to (epi)catechins), (epi)gallocatechins are expected to be stronger iron chelators than (epi)catechins (Figure S2). Jovanovic et al. found that the presence of an additional hydroxyl group in (epi)gallocatechins changed the iron chelation stoichiometry, where two molecules of (epi)gallocatechin, instead of three molecules of (epi)catechin, were required to chelate one ferrous ion at the physiological pH [58]. Thus, as tannin pea and chickpea seed coats contain exclusively (epi)gallocatechins (“G”-prodelphinidins”), whereas tannin faba bean, lentil, and black bean contain a mixture of (epi)catechins and (epi)gallocatechins, iron chelation is relatively stronger in pea and chickpea (see also Tables S9–S13). These findings suggest that the B-ring hydroxyl groups are expected to be mainly responsible for the iron chelation ability of polyphenols.

To illustrate the importance of proanthocyanidin composition, consider green (F3) and beige (F4) faba bean, dun (P4) pea and pinto (B4) common bean seed coat extracts that all showed similar proanthocyanidin content as estimated by the DMAC assay (Figure 3B) and antioxidant capacity (Figure 1), however, their iron chelation was quite different. As shown in Figure 8 and Table S11, proanthocyanidins in dun (P4) pea seed coat extract are “G”-prodelphinidins (i.e., containing almost exclusively (epi)gallocatechin), whereas green (F3) and beige (F4) faba bean seed coat extracts (Figure S4) contain procyanidins as well as both “G-C” and “G” prodelphinidins. In pinto (B4) common bean seed coats (Figure 6), both procyanidins and propelargonidins are observed [59]. Because (epi)afzelechin contains only one hydroxyl group in the B ring (Figure S2), (epi)afzelechin is hypothesized to be a weak iron chelator, as the presence of catechol (O-dihydroxyphenyl) or galloyl (trihydroxyphenyl) groups in a flavonoid are necessary to chelate iron [60]. Thus, the descending order of iron chelation ability for the above-mentioned extracts is dun (P4) pea > green (F3) ~ brown (F4) faba bean > pinto (B4) common bean.

Although differences in proanthocyanidin composition explained many of the differences in comparing iron chelation (Figure 4A) and the DMAC proanthocyanidin content (Figure 4B), it could not explain differences between black common bean seed coats and tannin lentils. The tannin lentils and black bean seed coats not only had similar proanthocyanidin profiles, but they also had similar amounts (Figure 4B). There are a couple of possible reasons to account for this difference. The first is that anthocyanins are highest in the black common bean and this might have contributed to a higher iron chelation. A more likely possibility is that there are large amounts of myricetin 3-*O*-glucoside (Table S4) found in black bean, whereas the tannin lentils contain mostly kaempferol glycosides. Myricetin 3-*O*-glucoside was shown to inhibit iron uptake in *Caco-2* cells, whereas kaempferol 3-*O*-glucoside was shown to promote uptake [12].

Thus, the results for these five pulse crop seed coat extracts suggest that the best indication of antioxidant potential and iron chelation ability is the concentration and type of proanthocyanidins, although other polyphenols, such as myricetin 3-*O*-glucoside, can also play a supporting role. These results can help to define the possible applications for these seed coats. For example, pinto (B4) common bean seed coats, rich in propelargonidins, could be incorporated in food matrices or supplements to boost their antioxidant properties or increase their shelf-life while minimizing unwanted effects of iron chelation after ingestion. Because of their high iron chelation ability, prodelphinidin-rich pea seed coats could be used to treat numerous iron overload-involved diseases [61]. Additionally, a recent study has demonstrated the successful use of a ferric-epigallocatechin 3-*O*-gallate complex as a microcapsule carrier for a hydrophobic anti-tuberculosis agent implicating potential use in pharmaceutical formulations [62]. The seed coats having high antioxidant activity could be added to food packaging films as a protective barrier against oxidation reactions and microbial growth. Additionally, these seed coats could be potential food-derived ingredients in cosmetic and skin care products, as prodelphinidin-rich extracts, such as grape seed extract, were reported to reduce skin hyperpigmentation and promote wound healing [63]. Recent research has shown a growing interest in the biological activities of proanthocyanidin-rich food byproducts as a renewable and environmentally friendly resource to be used in pharmaceutical, cosmetic and food applications [64].

## 3. Materials and Methods

### 3.1. Plant Material

Pulse crop production is established in current crop rotations of all agricultural zones in Western Canada. Seed coats of pulses are typically rich in polyphenols and display a wide range of colors, patterns and biochemical profiles. Our goal was to analyze seed coats across the widest possible range of phenotypes within the adapted germplasm base of the five pulse crops. We therefore selected characteristic genotypes from each of the five pulse crops (Table 3) grown in Western Canada and obtained from the Crop Development Centre at the University of Saskatchewan (Saskatoon, Canada). These seed genotypes were the same as used in our previous LC-MS study [26]. A white-flowered (white/grey seed coat) and a black seed coat variety (where available) of each pulse crop were included because they represent the lowest (i.e., low tannin) and highest polyphenol content, respectively. Low tannin genotypes express a similar gene that results in genetic blockage of part of the polyphenol pathway [65,66], whereas black varieties typically contain the highest amount of anthocyanins [67]. Two additional varieties with different seed coat colors, such as green, yellow or brown were included as these also show variability in composition [68]. The seeds were dehulled using an abrasive mill and the seed coats were separated using sieves and a column blower.

### 3.2. Chemicals and Reagents

A list of the chemicals and reagents used in this study along with supplier information is shown in Table S1.

### 3.3. Preparation of Seed Coat Extracts

Seed coat extracts were prepared using a procedure similar to that of Mirali et al. [69] and modified by Elessawy et al. [26]. Note that although the insoluble-bound phenolics have been shown to be abundant in pulse crops [70], only soluble polyphenols were extracted with this method. An important modification in this study was that internal standards used for targeted LC-MS were not added to the extraction solvent for either untargeted LC-MS (where quality control (QC) samples were used for relative quantification) or for the assays, as these polyphenol standards would affect the results of the assays. In brief, for all analyses, ∼200 mg of each sample was placed into a micro centrifuge tube that was covered, put in a −80 °C freezer for 1 h, and then freeze-dried overnight at −80 °C and less than 0.133 mbar using a FreeZone Plus 6 freeze dryer (LabConco, Kansas City, MO, USA). Two ¼ inch ceramic sphere beads were added to each tube and the seed coats were pulverized to a fine powder using a Mini-Beadbeater-16 (BioSpec Products, Inc., Bartlesville, OK, USA) for 30 s. A volume of 1 mL of the acetone:water (70:30 *v*/*v*) extraction solvent was added to the pulverized seed coats, and samples were mixed for 1 min using the Mini-Beadbeater-16, before being shaken for 1 h at 23 °C on a Thermomixer C (Eppendorf, Hamburg, Germany) at a speed of 1400 rpm. The samples were centrifuged at a speed of 16,200× *g* for 10 min, and each supernatant transferred into a new-labelled tube that was centrifuged at 16,200× *g* for 5 min a second time to ensure removal of all of the seed coat pellets. A 100 µL aliquot of each extract was transferred to a new Eppendorf tube, dried down in a CentriVap vacuum concentrator (LabConco, Kansas City, MO, USA), and reconstituted in 100 µL of MilliQ-water:methanol (90:10 *v*/*v*).

### 3.4. Ferrozine Iron Chelating Assay

A Ferrozine assay was used to measure the ability of seed coat extracts to chelate Fe^2+^ using the indirect colorimetric method reported by Carter [71] and modified by Santos et al. [31]. Seed coat extracts were diluted in 10% methanol as necessary to obtain absorbance readings in the linear range. Serial dilutions (1–50 µg/mL) of disodium ethylenediamine tetra-acetic acid (EDTA-Na_2_) were used to generate a standard curve. Distilled water and dilution solvent replaced the Ferrozine reagent and the seed coat sample in the blank and control samples, respectively. In a 96-well plate, 50 µL of each sample, control (solvent only), blank or standard (EDTA-Na_2_) solution were mixed with 160 µL of 50 mM ammonium acetate buffer (pH 6) and 20 µL of 0.3 mM FeSO_4_ solution. The plate was incubated for 5 min to allow ferrous chelation by polyphenols in the samples. A volume of 30 µL of 1 mM Ferrozine solution was added to each well to react with any free ferrous ions remaining in the reaction mixture forming a blue-colored complex that was monitored after 15 min at 562 nm in a microplate reader. A decrease in the absorbance (A) indicated an increase in iron chelating ability of the sample. Results are expressed as mg EDTA equivalents per mg dry weight of seed coat.

### 3.5. Antioxidant Capacity Assays

#### 3.5.1. DPPH Assay (Mixed-Mode HAT and ET Based)

The antioxidant capacity using a DPPH (1,1-diphenyl-2-picrylhydrazyl) assay was measured according to the method proposed by Brand-Williams et al. [72] and modified by Csepregi et al. [73]. An aliquot of 20 µL of each sample (the extract, the standard, or the solvent) was mixed with 180 µL of 0.5 mM DPPH methanolic solution in a microplate well, followed by a 30-min incubation at room temperature in a dark place. The plate was read in a microplate reader at 517 nm using myricetin 3-*O*-glucoside (50–450 µg/mL) as a standard to build the calibration curve [73]. Serial dilutions of each sample were prepared in methanol until they were in the calibration range. The percentage of DPPH scavenging activity was plotted against the sample/standard concentration to obtain an IC_50_ value, which represents the concentration of the extract or standard antioxidant (mg/mL) needed to scavenge 50% of the DPPH in the reaction mixture. As the IC_50_ value is inversely proportional to the antioxidant activity, the results are expressed in the form of the reciprocal of IC_50_, called the antiradical power (ARP, ARP = 1/IC_50_) [72].

#### 3.5.2. TBARS Assay

The TBARS (thiobarbituric acid reactive substances) assay estimated the concentration of malondialdehyde, a product of lipid peroxidation, as a measure of the antioxidant capability of polyphenols in the seed coat extracts to protect the lipid bilayer against peroxidation in liposomes as a simulated model for a biological cell membrane. This assay was conducted according to the method proposed by Subramanian et al. [74]. Preparation of liposomes [75,76] is described in the supplementary materials (Procedure S1). To measure the antioxidant activity of pulse seed coat extracts, 200 µL of liposome solution (1 mg/mL) was added to 40 µL of diluted extracts (10% methanol) and the mixture was shaken for 15 min. The control solution was prepared by mixing 10% methanol only with the liposome solution. Lipid oxidation was initiated by adding 40 µL of each of the following solutions: 0.5 mM FeSO_4_ solution, 5 mM ascorbic acid and water into the liposome-sample solution. The mixture was shaken and incubated in a 37 °C Thermomixer for 2 h. A volume of 40 µL of 3% sodium dodecyl sulfate and 400 µL of 0.375% TBA, 15% TCA, 0.25 M HCl (prepared by dissolving 0.375 g of thiobarbituric acid (TBA) and 15 g of trichloroacetic acid (TCA) in 100 mL of 0.25 M HCl solution) were added to the mixture, followed by vortexing and heating in a glycol bath at 95 °C for 30 min. After cooling, 800 µL of 1-butanol was added to each reaction mixture, vortexed for 20 s, and centrifuged at a speed of 16,200× *g* for 10 min. An aliquot of 200 µL of the organic (upper) layer was pipetted into a 96-well plate that was read using a microplate reader at 532 nm. Serial dilutions (2–52 µg/mL) of 1,1,3,3-tetramethoxypropane (TMP) in 10% methanol were used to construct a calibration curve for MDA [75]. An online IC_50_ calculator tool was used to calculate the concentration of each extract (µg/mL) required to inhibit 50% of the MDA formation (lipid peroxidation product) in the reaction mixture (IC_50_) [77]. Its reciprocal, the antiradical power (ARP, ARP = 1/IC_50_) was then calculated to facilitate comparing the results of different assays.

#### 3.5.3. Folin–Ciocalteu Assay (ET-Based)

The Folin–Ciocalteu assay used the method proposed by Ainsworth and Gillespie [78] and modified by Csepregi et al. [73]. In brief, samples were reconstituted and diluted 20 times using 10% methanol. An aliquot of 20 µL of each sample (extract, standard or solvent) was mixed with 40 µL of 10% Folin–Ciocalteu reagent and 160 µL of 0.7 M sodium carbonate solution in a microplate well. The plate was then incubated at room temperature for 2 h. Finally, the plate was measured in a microplate reader at 765 nm. Gallic acid (17–221 µg/mL) was used as a standard to build the calibration curve [73]. The antioxidant capacity of each sample was calculated using the linear equation of the calibration curve as mg gallic acid equivalent per gram of dry seed coat weight.

#### 3.5.4. Ferric Reducing Antioxidant Power (FRAP) Assay (ET-Based)

The ferric reducing antioxidant power (FRAP) of seed coat extracts was assessed using the method proposed by Benzie and Strain [79] and modified by Santos et al. [31]. The FRAP reagent was prepared by mixing 300 mM sodium acetate buffer (pH 3.6), 10 mM 2,4,6-Tripyridyl-s-triazine (TPTZ) solution in 40 mM HCl, and 20 mM FeCl_3_·6H_2_O using the proportion 10:1:1 (*v*/*v*/*v*). An aliquot of 290 µL of the freshly prepared FRAP reagent and 10 µL of each diluted sample was mixed in a 96-well plate. After a 30-min reaction time, the absorbance was read at λ = 593 nm. A standard curve with different concentrations of myricetin 3-*O*-glucoside (5–250 µg/mL) was created to calculate the ferric reducing antioxidant power of the samples. The results are expressed in mg myricetin 3-*O*-glucoside equivalents per mg dry weight of seed coat.

### 3.6. Spectrophotometric Measurement of Polymeric Polyphenols

Polymeric polyphenols (proanthocyanidins) were measured using the 4-dimethylaminocinnamaldehyde (DMAC) assay according to Wallace and Guisti [32]. Samples were diluted 100 times in methanol (except extracts of low tannin seed coats). Serial dilutions of procyanidin B_1_ (10–450 µg/mL) were prepared in methanol to construct a standard curve. In a 96-well plate, 5 µL of each standard, blank or diluted sample was mixed with 200 µL of methanol and 20 µL of 2% DMAC solution prepared in cold methanol:6N H_2_SO_4_ (1:1). This plate was incubated in a dark place at room temperature for 20 min. The absorbance was read at 640 nm in a microplate reader. Proanthocyanidin concentrations are reported as mg procyanidin B_1_ equivalents per mg dry weight of seed coat.

### 3.7. Untargeted Analysis of Seed Coat Extracts Using LC-HRMS

The LC-HRMS instrumentation consisted of a Dionex 3000 LC coupled with a Quadrupole-Orbitrap (Thermo Fisher Q-Exactive, Waltham, MA, USA) mass spectrometer with a HESI (heated ESI) source. For LC separation, an Agilent poroshell 120 PFP column (2.1 × 100 mm, 2.7 µm) was used at a flow rate of 0.35 mL/min. A 30 min run time was used and the mobile phases were water:formic acid (99.9:0.1, *v*/*v*) as solvent A, and water:acetonitrile:formic acid (9.9:90:0.1, *v*/*v*/*v*) as solvent B. After a one min hold at 1% B, gradient elution was performed according to the following conditions: from 1% B to 41% B in 20 min; 41% to 60% B in 4 min, 60% to 80% B in 0.1 min, hold at 80% B for 1.9 min, 80% to 1% B in 0.1 min, then hold at 1% B for 3.9 min. The Q-Exactive was used to acquire full scan data for the seed coat samples using a mass resolution (full width at half maximum, FWHM @ *m*/*z* 200) of 140,000 in negative mode and a mass range of 140–2100 *m*/*z*.

A QC (quality control) sample, which contains equal amounts of all 60 seed coat samples (four different colored seed coats of five pulse crops, with three replicates each), was injected every 8–10 runs to account for any change in retention time or signal intensity. Therefore, QC samples enable relative quantification for identified polyphenols. In addition, four ID (identification) samples, which contain an aliquot from all the samples within a color group across the selected pulse crops (one ID each for low tannin, black/maple, brown/beige, green/yellow seed coats), were prepared.

The ID samples were used to obtain fragmentation data using the scan function “Full scan/DDMS^2^”. DDMS^2^ (data dependent MS/MS) acquires fragmentation data on the most abundant ions detected in full-scan mode. Mass resolution of the full scan analysis was 70,000 (FWHM @ *m*/*z* 200) and MS/MS was carried out on the top (most abundant) 7 peaks at a resolution of 17,500 (FWHM @ *m*/*z* 200) from each scan using a stepped collision energy fragmentation (15, 35, and 55 eV). The MS/MS acquisition used an exclusion list (*m*/*z* values) of the most intense ions detected from the blank sample. Additional MS/MS spectra were also carried out at collision energies of 10 and 75 eV to assist in compound identification.

### 3.8. Data Analysis

**Figure 9:**
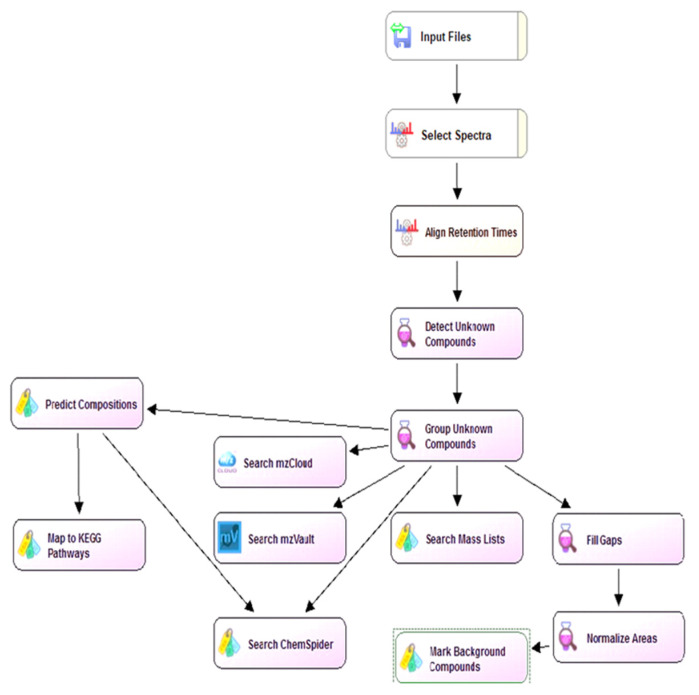
A flowchart showing the customized untargeted metabolomics workflow used in this study.

A customized untargeted workflow (Figure 9) was developed by adapting an existing workflow in the Compound Discoverer 3.1 software (Thermo Fisher) to process LC-HRMS raw data. The workflow is similar to one reported previously using Compound Discoverer 2.1 with some modifications [66]. In brief, raw data files are imported in the software (“Input Files” node) and spectra are selected in the “Select Spectra” node. In “Align Retention Times” node, alignment of retention time is performed based on an adaptive curve (using QC samples). We initially experienced some issues with alignment, but changing “Shift reference file” to “false” solved these issues. In the “Detect Unknown Compounds” node, ions (features) are extracted, and the likely chemical formulas are identified using isotope ratios and a mass tolerance of 5 ppm. Maximum element counts were set to C150 H300 N4 Na2 O180 P2 S2. The “Group Unknown Compounds” node performs adduct and isotopic peak grouping with a mass tolerance of 5 ppm and a RT tolerance of 0.2 min. “Fill Gaps” node determines missing areas values among the samples for each compound detected, when the intensity of a compound is below the detection threshold. The background ions are annotated and filtered in “Mark Background Compounds” node. The three nodes: “Fill Gaps”, “Normalize Areas” and “Mark Background Compounds” are used for relative quantification and to identify background compounds.

As numerous structural isomers can exist, fragment ions using MS/MS spectra are useful to help narrow down the possible identities. The unknown MS/MS spectra obtained from the HRMS analysis were compared with libraries of MS/MS spectra. “Search mzCloud”, is an online MS/MS library created by Thermo that contains ~19,000 compounds. “Search mzVault” is a user-created node for comparing MS/MS spectra with in-house standards or known unknowns. “Search Mass Lists” is another user-created node used to identify known polyphenols, based on a retention time and *m*/*z* list for any standards analyzed with the same LC-MS method. We used standards from our targeted method to help create mzVault and mass list libraries. The “ChemSpider Search” node searches for possible matches from databases based on *m*/*z* and chemical formula. A post-processing node “Differential Analysis” is used to find significant statistical differences between sample groups using interactive visualizations, such as volcano plots and heat maps. Available differential analyses are principal component analysis (PCA) and hierarchical cluster analysis (HCA). To focus on the more intense polyphenols, the results were filtered, which included using an area cut-off of 1.5B, and a retention time window between 2 and 17 min.

### 3.9. Statistics

Experiments were performed in triplicate and results are presented as arithmetic means ± SD. IBM SPSS Statistics software version 26 for Windows (SPSS, Inc., Armonk, NY, USA) was used to compare the antioxidant capacities of the seed coat extracts within each crop measured by the same assay. One-way analysis of variance (ANOVA) was performed and followed Tukey’s HSD post hoc test for multiple comparisons. Pearson correlation analyses were used to identify significant correlations between the antioxidant capacity measurements for polyphenol subclasses in the seed coat extracts. In all analyses, a *p*-value < 0.05 was considered statistically significant.

## 4. Conclusions

An LC-HRMS untargeted metabolomics approach was used to identify major polyphenols present in diverse genotypes of five pulse crops. The results show several polyphenols not previously identified by our targeted method (Table 2) and estimates of the amounts of various classes were given in Tables S9–S13.

The contribution of these major polyphenol classes to the antioxidant and iron chelation capabilities were subsequently explored. The antioxidant capacity was largely dictated by the contribution of proanthocyanidins, although anthocyanins and flavan-3-ols were also important. The iron chelation capability was highly dependent on the type of proanthocyanidins in the extracts. Prodelphinidins showed higher iron chelation ability compared with procyanidins, which in turn are higher than propelargonidins.

Based on these findings, seed coat extracts with high prodelphinidin content, such as maple (P2) and dun (P4) pea are the least desirable sources for natural antioxidants to be used in food applications. Conversely, although dun (P4) pea seed coats had a marginally higher antioxidant capacity compared with brown common bean (B4), the iron chelation was much less in brown common bean suggesting it is more suitable for food applications. The highest antioxidant capacities were found in the seed coats of colored lentil (L2, L3 and L4) and black (B2) common bean seed coats, although B2 had the highest iron chelation value, presumably due to myricetin 3-O-glucoside. Knowledge of the antioxidant activity of these compounds may enable plant breeders to select varieties that have less prodelphinidins (for example) to better balance the positive effects of antioxidant activity in diets with iron chelation effects. Alternatively, as prodelphinidins are strong iron chelators, they could have potential applications in treatment of iron-overload related diseases and removal of iron contaminants from wastewater. Further research is needed to assess the in vivo antioxidant and iron chelation abilities considering the bioavailability and metabolism of these polyphenolic extracts.

## Figures and Tables

**Table 1:** Pearson correlations among the measured assays and polyphenol subclasses previously measured in pulse seed coat extracts. Excluding the antioxidant capacity measured by the F-C assay, green-highlighted cells refer to the highest correlations between polyphenol classes and antioxidant capacity, orange-highlighted cells refer to the correlations of antioxidant capacity measured by different assays, blue-highlighted cells refer to the correlations between proanthocyanidin content and antioxidant capacity, and pink-highlighted cells refer to the correlation between proanthocyanidin content and iron chelation ability.

	DMAC	TBARS	F-C	DPPH	FRAP	Ferrozine
**Assays**						
DMAC ^P^	1	0.973 *	0.865 *	0.986 *	0.983 *	0.930 *
TBARS ^A^		1	0.831 *	0.979 *	0.982 *	0.943 *
F-C ^A^			1	0.849 *	0.832 *	0.846 *
DPPH ^A^				1	0.995 *	0.954 *
FRAP ^A^					1	0.966 *
Ferrozine ^C^						1
**Polyphenol classes**						
Anthocyanins	0.370 *	0.448 *	0.234	0.377 *	0.406 *	0.433 *
Dihydroflavonols	0.427 *	0.496 *	0.353 *	0.434 *	0.469 *	0.541 *
Flavones	0.521 *	0.537 *	0.402 *	0.499 *	0.550 *	0.526 *
Flavonols	−0.082	−0.104	−0.025	−0.115	−0.122	−0.157
Flavan-3-ols	0.771 *	0.722 *	0.560 *	0.744 *	0.716 *	0.593 *
Hydroxybenzoic acids	0.294 *	0.314 *	0.217	0.335 *	0.350 *	0.428 *
Hydroxycinnamic acids	0.337 *	0.299 *	0.413 *	0.264 *	0.260 *	0.269 *
Procyanidins	0.742 *	0.648 *	0.599 *	0.668 *	0.643 *	0.468 *

* Statistically significant at *p* < 0.05; ^A^ antioxidant assay; ^C^ iron chelation assay; ^P^ proanthocyanidin assay.

**Table 2:** Identification of the compounds used in the Hierarchal clustering plot (HCA). C: (epi)catechin, A: (epi)afzelechin and G: (epi)gallocatechin. (X) refers to the presence of an adduct, (Y) refers to the presence of a water adduct, and (Z) refers to the presence of a dimer. Identification levels are: confirmed (1), putative (2), isomeric (2/3), class only (3) and unidentified (4). Compounds in bold were (semi)quantified using the targeted method [26].

Name	Formula	Molecular Weight	RT [min]	Mass Error (ppm)	Identification Level
**Yellow Bean (B3)**					
Phenolic acid derivative	C11 H12 O6	240.06358	8.54	0.82	3
Phenolic acid derivative	C14 H24 O5	272.16243	10.59	0.21	3
Phenolic acid derivative	C14 H24 O5	272.1625	9.89	0.46	3
Afzelechin	C15 H14 O5	274.0845	10.29	1.38	2
Epiafzelechin	C15 H14 O5	274.08451	11.15	1.40	2
Phenolic acid deoxyhexoside	C15 H16 O10	356.07484	9.66	1.39	3
(Epi)afzelechin hexoside	C21 H24 O10	436.13738	7.70	1.00	2/3
**Kaempferol 3-*O*-glucoside (X, Z)**	**C21 H20 O11**	**448.10084**	**14.39**	**0.63**	**1**
**Quercetin 3-*O*-rhamnoside**	**C21 H20 O11**	**448.1013**	**14.715**	**1.65**	**1**
3,5-Dihydroxy-2-(4-hydroxyphenyl)-4-oxo-3,4-dihydro-2H-chromen-7-yl hexopyranoside	C21 H22 O11	450.11658	9.89	0.83	2/3
**Myricetin 3-*O*-rhamnoside**	**C21 H20 O12**	**464.09603**	**13.51**	**1.19**	**1**
Kaempferol acetyl hexoside	C23 H22 O12	490.11134	16.39	0.43	2/3
Kaempferol malonyl hexoside (X)	C24 H22 O14	534.10118	16.40	0.41	2/3
AA	C30 H26 O10	546.15358	11.42	1.80	2/3
AA	C30 H26 O10	546.1536	10.35	1.84	2/3
Kaempferol 3-O-sambioside (X)	C26 H28 O15	580.1433	13.41	0.82	2
Kaempferol dihexoside	C27 H30 O16	610.15424	12.47	1.39	2/3
AAC	C45 H38 O16	834.2176	10.94	1.93	2/3
**Brown bean (B4)**					
**(+)-Catechin**	**C15 H14 O6**	**290.07923**	**8.85**	**0.67**	**1**
(Epi)catechin hexoside	C21 H24 O11	452.13217	6.59	0.68	2/3
AC	C30 H26 O11	562.148	9.14	0.87	2/3
**Procyanidin B1**	**C30 H26 O12**	**578.14284**	**8.05**	**0.72**	**1**
CC (Z)	C30 H26 O12	578.14315	11.61	1.25	2/3
**Procyanidin B2**	**C30 H26 O12**	**578.14329**	**9.41**	**1.50**	**1**
ACC	C45 H38 O17	850.21255	10.25	1.94	2/3
ACC	C45 H38 O17	850.21255	9.91	1.94	2/3
CCC	C45 H38 O18	866.207	6.03	1.37	2/3
CCC	C45 H38 O18	866.20703	9.25	1.40	2/3
CCC	C45 H38 O18	866.20715	9.73	1.54	2/3
ACCC	C60 H50 O23	1138.27613	10.61	1.62	2/3
CCCC	C60 H50 O24	1154.27068	9.84	1.28	2/3
CCCC	C60 H50 O24	1154.2707	9.97	1.30	2/3
CCCC	C60 H50 O24	1154.27127	11.69	1.80	2/3
ACCCC	C75 H62 O29	1426.3409	11.45	2.26	2/3
CCCCC	C75 H62 O30	1442.33558	10.99	2.07	2/3
CCCCC	C75 H62 O30	1442.33568	10.45	2.14	2/3
CCCCCC	C90 H74 O36	1730.39828	11.68	1.33	2/3
CCCCCCC	C105 H86 O42	2018.46508	12.00	2.83	2/3
**Black bean (B2)**					
**(−)-Gallocatechin**	**C15 H14 O7**	**306.07417**	**6.89**	**0.71**	**1**
**Vanillic acid 4- β -d-glucoside**	**C14 H18 O9**	**330.0953**	**5.632**	**0.66**	**1**
**Delphinidin 3- β -d-Glucoside (myrtillin) (Y)**	**C21 H20 O12**	**464.0966**	**6.47**	**2.43**	**1**
(Epi)gallocatechin hexoside	C21 H24 O12	468.12707	4.85	0.62	2/3
Myricetin 3-*O*-glucoside	C21 H20 O13	480.09095	12.36	1.17	2
Quercetin hexoside derivative	C24 H26 O13	522.13791	9.48	1.10	2/3
Quercetin hexoside derivative	C24 H26 O13	522.13792	10.21	1.11	2/3
GC	C30 H26 O13	594.13794	6.68	1.00	2/3
GG	C30 H26 O14	610.13304	4.82	1.28	2/3
Phenolic acid dihexoside derivative	C28 H34 O18	658.17597	6.47	2.22	3
Phenolic acid dihexoside derivative	C29 H36 O18	672.19118	7.75	1.51	3
Soyasaponin A1 (X)	C59 H96 O29	1268.60579	16.05	1.62	2
GGCCC	C75 H62 O32	1474.32534	9.09	1.98	2/3
GGGGC	C75 H62 O34	1506.31495	7.968	1.79	2/3
**Lentil (L2, L3 and L4)**					
Pantothenic acid	C9 H17 N O5	219.1107	3.04	0.13	2
Thymidine	C10 H14 N2 O5	242.0905	3.44	0.96	2
Hydroxybenzoic acid hexoside	C13 H16 O8	300.08462	6.40	0.36	2/3
Hydroxybenzoic acid hexoside	C13 H16 O8	300.08472	3.94	0.68	2/3
Hydroxybenzoic acid derivative	C16 H20 O11	388.10104	9.22	1.23	3
5-*O*-[β-apiosyl-(1-2)-*O*-β-xylopyranosyl]gentisic acid	C17 H22 O12	418.11142	10.98	0.71	2
Dihydroxybenzoic acid pentoside hexoside	C18 H24 O13	448.12215	9.43	1.03	2/3
Phenolic acid derivative	C21 H32 O13	492.18471	12.43	0.84	3
**Procyanidin B3**	**C30 H26 O12**	**578.14298**	**8.71**	**0.95**	**1**
CC	C30 H26 O12	578.14298	10.40	0.96	2/3
GC	C30 H26 O13	594.13772	8.67	0.64	2/3
GC (Z)	C30 H26 O13	594.13794	7.53	1.00	2/3
GG (Z)	C30 H26 O14	610.13321	6.48	1.57	2/3
CCC	C45 H38 O18	866.20698	10.87	1.35	2/3
GCC (Z)	C45 H38 O19	882.20148	9.54	0.86	2/3
GCC	C45 H38 O19	882.20187	8.24	1.29	2/3
GGC	C45 H38 O20	898.19641	7.34	0.86	2/3
GGC (Z)	C45 H38 O20	898.19674	9.98	1.22	2/3
GGG	C45 H38 O21	914.19169	6.63	1.24	2/3
GCCC	C60 H50 O25	1170.26587	9.38	1.50	2/3
GGCC	C60 H50 O26	1186.261455	8.48	2.04	2/3
GGCC	C60 H50 O26	1186.26057	9.66	1.30	2/3
GGGC	C60 H50 O27	1202.25548	7.85	1.27	2/3
GCCCC	C75 H62 O31	1458.33039	10.23	1.98	2/3
GGCCC	C75 H62 O32	1474.32422	10.53	1.22	2/3
GGCCC	C75 H62 O32	1474.32441	9.35	1.35	2/3
GGGCC	C75 H62 O33	1490.31944	8.78	1.42	2/3
GGGCC	C75 H62 O33	1490.31961	9.02	1.53	2/3
GGGGC	C75 H62 O34	1506.31474	8.12	1.65	2/3
GGGCCC	C90 H74 O39	1778.38314	9.71	1.36	2/3
GGGGCC	C90 H74 O40	1794.37975	9.19	2.29	2/3
GGGGGC	C90 H74 O41	1810.37349	8.47	1.62	2/3
GGGCCCC	C105 H86 O45	2066.4502	10.55	2.95	2/3
GGGGCCC	C105 H86 O46	2082.44459	9.92	2.67	2/3
**Green lentil (L3)**					
Quercetin pentoside	C17 H22 O13	434.10639	14.31	0.80	2/3
**Catechin 3-*O*-glucoside (Z)**	**C21 H24 O11**	**452.13219**	**8.02**	**0.73**	**2**
**Kaempferol di-rutinoside**	**C39 H50 O24**	**902.27023**	**10.11**	**1.14**	**2/3**
**Brown lentil (L4)**					
Phenolic acid derivative	C13 H20 O4	240.13636	12.97	0.85	3
Trihydroxy-megastigmadien-one hexoside	C19 H30 O9	402.18923	11.57	0.61	2/3
Trihydroxy-megastigmadien-one hexoside (Z)	C19 H30 O9	402.18928	11.47	0.75	2/3
Trihydroxy-megastigma-en-one hexoside	C19 H32 O9	404.20496	10.20	0.80	2/3
**Black lentil (L2)**					
Quercetin deoxyhexoside	C21 H20 O11	448.10092	16.75	0.79	2/3
**Luteolin 4′-*O*-glucoside**	**C21 H20 O11**	**448.10104**	**16.14**	**1.07**	**1**
Tricetin hexoside	C21 H20 O12	464.09605	16.75	1.24	2/3
Tricetin hexoside	C21 H20 O12	464.09606	15.16	1.26	2/3
Phenolic acid derivative	C20 H28 O14	492.14852	7.91	1.26	3
Delphinidin 3-*O*-(2-*O*-β-d-Glucopyranosyl-α-l-arabinopyranoside) (Y)	C26 H28 O16	596.13879	6.68	1.77	2
GCC	C45 H38 O19	882.2021	8.36	1.55	2/3
GGC	C45 H38 O20	898.19622	7.43	0.64	2/3
GGCC	C60 H50 O26	1186.26075	8.56	1.45	2/3
GGGGC	C75 H62 O34	1506.31576	8.30	2.33	2/3
**Pea (P2 and P4)**					
**3,4-Dihydroxybenzoic acid**	**C7 H6 O4**	**154.02662**	**5.59**	**0.07**	**1**
Aspartic acid derivative	C9 H15 N O5	217.09494	5.95	−0.36	3
*N*-phenylacetyl-aspartic acid	C12 H13 N O5	251.07947	9.26	0.39	2
*N*-salicyloyl-aspartic acid	C11 H11 N O6	253.05873	7.28	0.35	2
Aspartic acid derivative	C11 H17 N O7	275.10071	6.55	0.76	3
*N*-coumaroyl-aspartic acid isomer	C13 H13 N O6	279.07431	10.45	0.08	2/3
*N*-coumaroyl-aspartic acid isomer	C13 H13 N O6	279.07438	8.80	0.32	2/3
*N*-[(2,4-Dihydroxyphenyl)acetyl]-l-aspartic acid	C12 H13 N O7	283.0693	8.31	0.34	2
Amino acid derivative	C13 H15 N O7	297.08486	7.21	0.03	3
*N*-feruloyl-aspartic acid isomer	C14 H15 N O7	309.0849	9.73	0.17	2/3
*N*-feruloyl-aspartic acid isomer	C14 H15 N O7	309.08498	11.31	0.41	2/3
*N*-(2,4,6-Trimethoxybenzyl)-l-aspartic acid	C14 H19 N O7	313.11643	11.49	0.88	2
Aspartic acid derivative	C14 H21 N O7	315.13213	10.91	1.05	3
Aspartic acid derivative	C13 H21 N O9	335.12185	5.48	0.66	3
Gibberellic acid or isomer	C19 H22 O6	346.14182	10.99	0.53	2/3
Unidentified		404.13211	3.81		4
Phenolic acid derivative	C18 H28 O10	404.16868	8.59	1.07	3
Unidentified		406.1843	8.75		4
Amino acid derivative	C17 H21 N O11	415.11196	8.96	1.21	3
Aspartic acid derivative	C20 H24 N2 O11	468.13798	6.78	−0.06	3
Aspartic acid derivative	C20 H24 N2 O11	468.13798	5.64	−0.05	3
*N*-[[3-(**β** -d-Glucopyranosyloxy)-2,3-dihydro-2-oxo-1H-indol-3-yl]acetyl]aspartic acid	C20 H24 N2 O12	484.13328	4.19	0.74	2
Chlorinated aspartic acid derivative	C21 H27 Cl N2 O6 S2	502.09938	8.65	−1.06	3
Chlorinated aspartic acid derivative (Z)	C21 H27 Cl N2 O6 S2	502.09938	7.45	−1.05	3
Chlorinated aspartic acid derivative	C21 H27 Cl N2 O7 S2	518.09414	5.23	−1.31	3
Unidentified		589.27425	11.76		4
GG	C30 H26 O14	610.13302	6.17	1.25	2/3
GG (Z)	C30 H26 O14	610.13303	8.24	1.27	2/3
GGG	C45 H38 O21	914.1916	7.72	1.14	2/3
GGG	C45 H38 O21	914.19184	4.37	1.40	2/3
GGG	C45 H38 O21	914.19192	7.82	1.49	2/3
GGGG	C60 H50 O28	1218.25088	9.58	1.66	2/3
GGGGG	C75 H62 O35	1522.3086	10.05	0.95	2/3
GGGGGG	C90 H74 O42	1826.36757	8.90	1.15	2/3
GGGGGGG	C105 H86 O49	2130.43022	9.17	3.03	2/3
**Chickpea (C2, C3 and C4)**					
Dihydroxybenzoic acid	C7 H6 O4	154.02667	10.99	0.39	3
**Gallic acid**	**C7 H6 O5**	**170.02166**	**3.74**	**0.80**	**1**
Glutamyl phenylalanine	C14 H18 N2 O5	294.12176	6.12	0.64	2
Glutamyl tyrosine	C14 H18 N2 O6	310.11664	4.61	0.48	2
Dihydroxybenzoic acid hexoside	C13 H16 O9	316.07957	8.48	0.43	2/3
Dihydroxybenzoic acid hexoside	C13 H16 O9	316.07964	8.97	0.65	2/3
Trihydroxybenzoic acid hexoside	C13 H16 O10	332.07459	13.38	0.72	2/3
11-hydroxy-9,10-dihydrojasmonic acid 11-β-d-glucoside	C18 H30 O9	390.18933	7.14	0.88	2
Unidentified		396.16328	6.18		4
Hydroxybenzoic acid hexoside pentoside	C18 H24 O12	432.12679	5.74	0.04	2/3
**Kaempferol 3-*O*-rutinoside (Nicotiflorin)**	**C27 H30 O15**	**594.15915**	**13.95**	**1.15**	**1**
**Quercetin 3-*O*-rutinoside (Rutin) (X)**	**C27 H30 O16**	**610.15399**	**13.03**	**1.00**	**1**
Myricetin hexoside deoxyhexoside	C27 H30 O17	626.14864	12.19	0.55	2/3
Myricetin hexoside deoxyhexoside	C27 H30 O17	626.14869	12.11	0.63	2/3
Kaempferol pentoside-hexoside-deoxyhexoside	C32 H38 O19	726.20143	12.95	0.96	2/3
Quercetin pentoside-hexoside-deoxyhexoside	C32 H38 O20	742.19611	12.15	0.62	2/3
Quercetin pentoside-hexoside-deoxyhexoside	C32 H38 O20	742.19625	12.24	0.81	2/3
GG-deoxyhexoside	C33 H40 O20	756.21216	13.14	1.15	2/3
Myricetin pentoside-hexoside-deoxyhexoside	C32 H38 O21	758.19094	11.32	0.50	2/3
Myricetin pentoside-hexoside-deoxyhexoside	C32 H38 O21	758.19098	11.55	0.55	2/3
Myricetin hexoside dideoxyhexoside	C33 H40 O21	772.20664	12.29	0.56	2/3
Myricetin derivative	C34 H42 O22	802.21764	11.20	1.08	3
GGGG	C60 H50 O28	1218.2508	7.33	1.60	2/3
GGGGG	C75 H62 O35	1522.30948	7.66	1.52	2/3
**Faba bean (F2, F3 and F4)**					
Phenolic acid derivative	C11 H12 O5	224.06867	10.99	0.89	3
Hydroxyjasmonic acid	C12 H18 O4	226.12081	8.65	1.35	2
Prolyl aspartic acid	C9 H14 N2 O5	230.09031	5.48	0.16	2
Phenolic acid derivative	C12 H16 O5	240.10005	9.19	1.14	3
Phenolic acid derivative	C12 H18 O5	242.1157	5.86	1.15	3
Phenolic acid derivative	C12 H18 O6	258.11044	5.42	0.39	3
Phenolic acid derivative	C12 H18 O6	258.11047	6.24	0.51	3
Phenolic acid derivative	C12 H14 O7	270.07392	9.62	−0.14	3
**(-)-Epicatechin**	**C15 H14 O6**	**290.07922**	**10.04**	**0.64**	**1**
**(-)-Epigallocatechin**	**C15 H14 O7**	**306.0742**	**8.49**	**0.80**	**1**
Aspartic acid derivative	C14 H14 N2 O7	322.08015	6.47	0.16	3
Aspartic acid derivative (Z)	C14 H14 N2 O7	322.08016	4.75	0.18	3
Phenolic acid derivative	C15 H18 N O9 P	387.07262	12.08	1.81	3
Phenolic acid derivative	C18 H28 O9	388.17373	7.18	1.04	3
Phenolic acid derivative	C18 H28 O10	404.1687	11.13	1.11	3
Unidentified		427.22128	13.11		4
Caffeic acid malonyl hexoside (X)	C16 H28 O13	428.15318	2.24	0.44	2/3
(Epi)catechin hexoside	C21 H24 O11	452.13245	9.34	1.31	2/3
(Epi)gallocatechin hexoside	C21 H24 O12	468.12722	7.88	0.95	2/3
*N*-[[3-(**β** -d-Glucopyranosyloxy)-2,3-dihydro-2-oxo-1H-indol-3-yl]acetyl]aspartic acid	C20 H24 N2 O12	484.13314	4.03	0.45	2
CC (Z)	C30 H26 O12	578.14271	9.83	0.50	2/3
GC	C30 H26 O13	594.13794	7.22	1.01	2/3
GC	C30 H26 O13	594.13798	6.32	1.08	2/3
GC	C30 H26 O13	594.13803	9.01	1.16	2/3
Unidentified		594.15926	10.25		4
GCC	C45 H38 O19	882.20213	5.17	1.59	2/3
GGC	C45 H38 O20	898.19605	4.78	0.45	2/3

**Table 3:** Detailed description of pulse crops genotypes used in this study.

Pulse Crop	Sample Code	Seed Coat Genotype	Seed Coat Color	High/Low Tannin	Seed Pictures
**Chickpea** **(*Cicer arietinum* L.)**	C1	CDC Xena	White	Low tannin	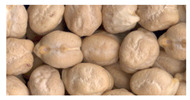
	C2	CDC Ebony	Black	High tannin	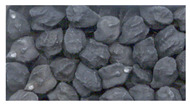
	C3	CDC Jade	Green	High tannin	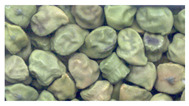
	C4	CDC Cory	Brown	High tannin	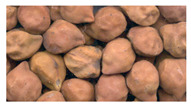
**Faba bean** **(*Vicia faba* L.)**	F1	CDC Snowdrop	White	Low tannin	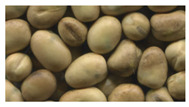
	F2	Black Fava	Black	High tannin	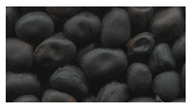
	F3	Masterpiece	Green	High tannin	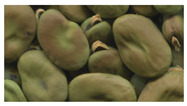
	F4	749-13-2015	Beige	High tannin	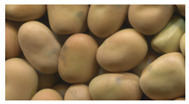
**Lentil** **(*Lens culinaris* Medik.)**	L1	6500ZT-4	Grey	Low tannin	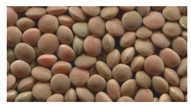
	L2	Indianhead	Black	High tannin	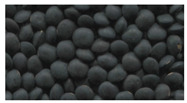
	L3	CDC Kermit	Green	High tannin	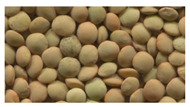
	L4	CDC Robin	Brown	High tannin	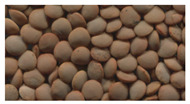
**Pea** **(*Pisum sativum* L.)**	P1	CDC Meadow	White	Low tannin	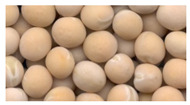
	P2	30/2-ILT	Maple (patterned)	High tannin	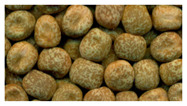
	P3	MFR042	Green	Low tannin	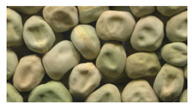
	P4	CDC Dakota	Dun (brown)	High tannin	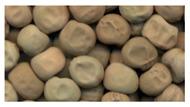
**Common bean** **(*Phaseolus vulgaris* L.)**	B1	Envoy	White	Low tannin	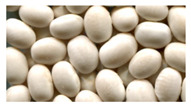
	B2	CDC Jet	Black	High tannin	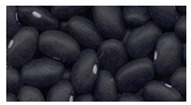
	B3	CDC Sol	Yellow	High tannin	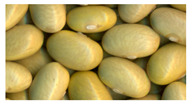
	B4	CDC WM-2	Brown (pinto)	High tannin	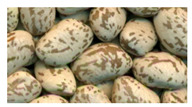

## Data Availability

The data presented in this study are available on request from the corresponding author.

## Supplementary Materials

The following are available online. Procedure S1: Liposome preparation, Figure S1: General structure of monomeric and examples of polymeric flavonoids, Figure S2: Basic structure of flavonoid rings (X), and flavan-3-ol monomers (Y and Z) that polymerize to form different types of proanthocyanidin polymers, Figure S3: A Principal component analysis (PCA) plot of PC1 versus PC2 of faba bean (A), lentil (B) and common bean (C) seed coats, Figure S4: HRMS full scan showing a procyanidin pentamer and different prodelphinidin pentamers detected in faba bean seed coats, Table S1: List of chemicals/reagents used in this study and their suppliers, Table S2: Antioxidant capacity measured for pulse seed coat extracts using four assays, Table S3: Proanthocyanidin content and iron chelation ability of pulse seed coat extracts. Table S4. Concentrations of polyphenols in common bean seed coat extracts (µmol/g dry weight), Table S5. Concentrations of polyphenols in lentil seed coat extracts (µmol/g dry weight), Table S6. Concentrations of polyphenols in pea seed coat extracts (µmol/g dry weight), Table S7. Concentrations of polyphenols in chickpea seed coat extracts (µmol/g dry weight), Table S8. Concentrations of polyphenols in faba bean seed coat extracts (µmol/g dry weight), Table S9. Estimated amounts (µmol/g) of major polyphenols detected in common bean seed coats by the untargeted method and not quantified in Table S4, Table S10. Estimated amounts (µmol/g) of major polyphenols detected in lentil seed coats by the untargeted method and not quantified in Table S5, Table S11. Estimated amounts (µmol/g) of major polyphenols detected in pea seed coats by the untargeted method and not quantified in Table S6, Table S12. Estimated amounts (µmol/g) of major polyphenols detected in pea seed coats by the untargeted method and not quantified in Table S7, Table S13. Estimated amounts (µmol/g) of major polyphenols detected in faba bean seed coats by the untargeted method and not quantified in Table S8.
