# Supplementary material for: An Untargeted Metabolomics Approach for Correlating Pulse Crop Seed Coat Polyphenol Profiles with Antioxidant Capacity and Iron Chelation Ability
Source: Molecules. 2021 Jun 23;26(13):3833. doi: 10.3390/molecules26133833 (PMC8270320; doi:10.3390/molecules26133833)
Supplement: Supplementary file 1 [file molecules-26-03833-s001.zip › molecules-1262975-supplementary.pdf]

Supplementary materials

Procedures

Procedure S_1: Liposome preparation

Empty liposomes were prepared from soy phosphatidyl choline (PC) by dissolving 200 mg of PC in 50 mL of ethyl acetate to dissolve the lipid [75,76]. Ethyl acetate was evaporated in a rotary evaporator to get a uniform thin dried lipid layer, which was re-dissolved in 100 mL of warm water and sonicated for 1 h at 55°C to form unilamellar vesicles (liposomes, particle size measured by Malvern zetasizer was 115 ± 15 nm). A volume of warm water (100 mL) was then added to the liposome solution bringing its final concentration to 1 mg/mL.

Tables

Table S_1. List of chemicals/reagents used in this study and their suppliers

Chemical name	Supplier
1,1,3,3-tetramethoxypropane	Sigma Aldrich, US
1-butanol	Sigma Aldrich, US
2,4,6-Tripyridyl-S-triazine (TPTZ)	Sigma Aldrich, US
2-thiobarbituric acid	Sigma Aldrich, US
4-(dimethylamino) cinnamaldehyde (DMAC)	Sigma Aldrich, US
Ammonium acetate	Sigma Aldrich, US
Ascorbic acid	Fisher Scientific, Canada
EDTA-Na_2	Sigma Aldrich, US
Ferric chloride hexahydrate	Sigma Aldrich, US
Ferrozine (3-(2-pyridyl)-5,6-diphenyl-1,2,4-triazine-4',4''-disulfonic acid sodium salt)	Sigma Aldrich, US
Folin Ciocalteu reagent	Sigma Aldrich, US
Gallic acid	Sigma Aldrich, US
Hydrochloric acid	EMD Serono, Canada
Iron (II) sulfate heptahydrate	Sigma Aldrich, US
Methanol	Fisher Chemical, US
Myricetin 3-O-glucoside	Extrasynthese, France
Phospahtidyl choline (soy 95%)	Sigma Aldrich, US
Procyanidin B1	Sigma Aldrich, US
Sodium acetate	Sigma Aldrich, US
Sodium carbonate	Sigma Aldrich, US
Sodium dodecyl sulfate	Sigma Aldrich, US
Sulfuric acid	Fisher Scientific, Canada
Trichloroacetic acid (TCA)	Sigma Aldrich, US

Table S$_2$. Antioxidant capacity measured for pulse seed coat extracts using four assays.

Pulse crop	Sample code	Seed coat color	Antioxidant capacity			
			F-C assay**	DPPH assay (ARP)***	FRAP****	TBARS*****
**Chickpea (*Cicer arietinum* L.)**	C1	White*	2.84 ± 0.59[a]	0.30 ± 0.04[a]	0.19 ± 0.01[a]	0.020 ± 0.015[a]
	C2	Black	13.3 ± 0.2[d]	156 ± 1[e]	20.5 ± 0.2[g]	0.20 ± 0.01[d]
	C3	Green	9.76 ± 0.79[b]	111 ± 3[d]	13.6 ± 0.4[f]	0.16 ± 0.00[d]
	C4	Brown	11.9 ± 0.7[c,d]	161 ± 9[e]	18.4 ± 0.6[f,g]	0.17 ± 0.03[d]
**Faba bean (*Vicia faba* L.)**	F1	White*	2.72 ± 0.31[a]	11.0 ± 0.9[b]	1.26 ± 0.01[c]	0.05 ± 0.02[b]
	F2	Black	24.1 ± 0.3[e,f]	522 ± 5[h]	59.5 ± 1.0[i,j]	0.30 ± 0.01[e,f]
	F3	Green	23.1 ± 0.4[e]	430 ± 4[g]	56.1 ± 1.5[i]	0.27 ± 0.00[e]
	F4	Beige	24.4 ± 0.8[f,g]	510 ± 1[h]	64.9 ± 2.5[j]	0.35 ± 0.02[f]
**Lentil (*Lens culinaris* Medik.)**	L1	Grey*	3.76 ± 1.08[a]	13.9 ± 0.1[b]	1.53 ± 0.00[d]	0.077 ± 0.003[c]
	L2	Black	25.2 ± 0.1[g,h]	962 ± 16[j]	145 ± 5[m]	0.66 ± 0.01[h]
	L3	Green	25.1 ± 0.3[f,g,h]	1136 ± 11[l]	144 ± 2[m]	0.66 ± 0.04[h]
	L4	Brown	24.8 ± 0.1[f,g,h]	1189 ± 9[m]	157 ± 3[n]	0.61 ± 0.03[h]
**Pea (*Pisum sativum* L.)**	P1	White*	3.94 ± 0.70[a]	2.90 ± 0.07[a]	0.95 ± 0.03[b]	0.023 ± 0.003[a]
	P2	Maple (patterned)	25.8 ± 0.1[h]	880 ± 16[i]	121 ± 4[l]	0.50 ± 0.02[g]
	P3	Green marrowfat	4.35 ± 1.57[a]	1.10 ± 0.05[a]	0.29 ± 0.01[a]	0.007 ± 0.000[a]
	P4	Dun (brown)	25.4 ± 0.2[g,h]	531 ± 3[h]	78.8 ± 3.1[k]	0.29 ± 0.05[e,f]
**Common bean (*Phaseolus vulgaris* L.)**	B1	White*	3.36 ± 0.16[a]	0.40 ± 0.04[a]	0.03 ± 0.00[a]	0.006 ± 0.001[a]
	B2	Black	25.4 ± 0.4[g,h]	1091 ± 25[k]	156 ± 3[n]	0.73 ± 0.01[i]
	B3	Yellow	11.5 ± 0.4[b,c]	51.0 ± 0.7[c]	4.63 ± 0.19[e]	0.069 ± 0.003[b,c]
	B4	Pinto (patterned)	23.8 ± 0.2[e]	309 ± 5[f]	32.5 ± 0.9[h]	0.19 ± 0.02[d]

*Low tannin seed coats

**F-C: Folin Ciocalteau assay; F-C was estimated as mg gallic acid equivalent/g dry weight of seed coat

***ARP: antiradical power; APR is the reciprocal of IC_{50}. All values were multiplied by 10^{-3} for simplicity

****FRAP: Ferric reducing antioxidant power was estimated as mg myricetin 3-O-glucoside equivalent/g dry weight of seed coat

*****TBARS: Thiobarbituric acid reactive substances assay was estimated as the reciprocal of IC_{50} values. Results were expressed as mean ± SD. All concentrations were calculated based on dry weight. Means with different superscript letters for each assay are significantly different ($p < 0.05$).

Table S3. Proanthocyanidin content and iron chelation ability of pulse seed coat extracts

Pulse crop	Sample code	Seed coat color	Proanthocyanidin content**	Iron chelation ability***
**Chickpea (*Cicer arietinum* L.)**	C1	White*	0.01 ± 0.00[a]	0.10 ± 0.01[a]
	C2	Black	6.16 ± 0.06[c]	21.8 ± 0.5[f]
	C3	Green	9.70 ± 0.06[d]	15.8 ± 0.3[e]
	C4	Brown	10.2 ± 0.1[d]	19.7 ± 0.1[e,f]
**Faba bean (*Vicia faba* L.)**	F1	White*	0.01 ± 0.00[a]	0.23 ± 0.01[b]
	F2	Black	24.5 ± 0.7[e]	45.7 ± 0.8[g]
	F3	Green	34.7 ± 0.6[f]	43.0 ± 3.2[g]
	F4	Beige	40.4 ± 0.7[g]	45.2 ± 0.7[g]
**Lentil (*Lens culinaris* Medik.)**	L1	Grey*	0.53 ± 0.01[b]	0.86 ± 0.02[c]
	L2	Black	70.3 ± 2.8[i]	89.2 ± 4.7[i]
	L3	Green	76.8 ± 1.0[k]	89.1 ± 0.8[i]
	L4	Brown	75.6 ± 0.6[j,k]	91.3 ± 1.3[i]
**Pea (*Pisum sativum* L.)**	P1	White*	0.00 ± 0.00[a]	0.10 ± 0.00[a]
	P2	Maple (patterned)	55.1 ± 0.3[h]	110 ± 4[j]
	P3	Green marrowfat	0.00 ± 0.00[a]	0.06 ± 0.03[a]
	P4	Dun (brown)	34.7 ± 0.2[f]	81.1 ± 4.4[h]
**Common bean (*Phaseolus vulgaris* L.)**	B1	White*	0.01 ± 0.00[a]	0.08 ± 0.01[a]
	B2	Black	72.8 ± 0.5[i,j]	116 ± 1[j]
	B3	Yellow	5.99 ± 0.06[c]	2.12 ± 0.02[d]
	B4	Pinto (patterned)	32.3 ± 0.5[f]	15.0 ± 0.4[e]

*Low tannin seed coats

**Proanthocyanidin content was estimated by DMAC assay as mg procyanidin B_1 equivalent/g dry weight of seed coat

***Iron chelation ability was estimated by Ferrozine assay as mg EDTA equivalent/g dry weight of seed coat

All concentrations were calculated based on dry weight. Means with different superscript letters for each assay are significantly different ($p < 0.05$)

Table S4. Concentrations of polyphenols in common bean seed coat extracts (µmol/g dry weight)

Compound	B1			B2			B3			B4		
	Mean	±	SD	Mean	±	SD	Mean	±	SD	Mean	±	SD
Pelargonidin 3-O-glucoside	ND	±	ND	1.75	±	0.23	ND	±	ND	ND	±	ND
Cyanidin 3,5-di-O-glucoside	ND	±	ND	13.8	±	0.8	ND	±	ND	ND	±	ND
Delphinidin 3,5-di-O-glucoside	ND	±	ND	198	±	31	ND	±	ND	ND	±	ND
Delphinidin 3-O-rhamnoside	ND	±	ND	1.73	±	0.23	ND	±	ND	ND	±	ND
Delphinidin 3-β-D-Glucoside	ND	±	ND	4873	±	1509	ND	±	ND	ND	±	ND
Cyanindin 3-O-glucoside	ND	±	ND	51.1	±	3.2	ND	±	ND	ND	±	ND
Malvidin 3,5-di-O-glucoside	ND	±	ND	305	±	21	ND	±	ND	ND	±	ND
Malvidin 3-O-galactoside	ND	±	ND	15.2	±	1.5	ND	±	ND	ND	±	ND
Malvidin 3-O-glucoside	ND	±	ND	405	±	25	ND	±	ND	ND	±	ND
Peonidin 3,5-di-O-glucoside	ND	±	ND	1.13	±	0.08	ND	±	ND	ND	±	ND
Peonidin 3-O-glucoside	ND	±	ND	3.90	±	0.64	ND	±	ND	ND	±	ND
Anthocyanins	ND	±	ND	5870	±	1592	ND	±	ND	ND	±	ND
Chalcones	ND	±	ND	ND	±	ND	ND	±	ND	ND	±	ND
Dihydromyricetin	ND	±	ND	151	±	12	ND	±	ND	ND	±	ND
Dihydrokaempferol	ND	±	ND	6.35	±	0.37	15.0	±	1.5	2.27	±	0.36
Taxifolin (dihydroquercetin)	ND	±	ND	14.2	±	0.7	7.42	±	0.74	1.37	±	0.05
Dihydroflavonols	ND	±	ND	172	±	13	22.4	±	2.3	3.64	±	0.41
Luteolin 3',7-di-O-glucoside	ND	±	ND	ND	±	ND	0.40	±	0.10	ND	±	ND
Flavones	ND	±	ND	ND	±	ND	0.40	±	0.10	ND	±	ND
Kaempferol	ND	±	ND	9.62	±	1.92	280	±	48	60.1	±	17.8
Kaempferol 3-O-D-galactoside	ND	±	ND	ND	±	ND	0.96	±	0.26	ND	±	ND
Kaempferol 3-O-glucoside	0.75	±	0.10	837	±	64	8877	±	472	1892	±	158
Kaempferol 3-O-rutinoside	0.31	±	0.08	1.06	±	0.60	ND	±	ND	0.17	±	0.02

Kaempferol 7-O-glucoside	ND ± ND	0.57 ± 0.13	23.3 ± 7.2	3.79 ± 0.31
Kaempferol di-rutinoside	0.24 ± 0.03	1.53 ± 0.45	0.30 ± 0.08	0.18 ± 0.02
Myricetin	ND ± ND	82.5 ± 23.4	ND ± ND	ND ± ND
Quercetin	ND ± ND	14.5 ± 3.0	21.2 ± 4.8	0.75 ± 0.14
Quercetin 3,4'-di-O-glucoside	ND ± ND	0.77 ± 0.05	1.55 ± 0.27	ND ± ND
Quercetin 3-O-glucoside (Isoquercetrin)	ND ± ND	287 ± 29	455 ± 23	7.95 ± 0.92
Quercetin 3-O-rutinoside(Rutin)	ND ± ND	0.99 ± 0.13	ND ± ND	0.15 ± 0.04
Quercetin 4′-O-glucoside (Spiraeoside)	ND ± ND	0.33 ± 0.05	15.8 ± 4.5	ND ± ND
Flavonols	1.30 ± 0.21	1236 ± 123	9675 ± 560	1965 ± 177
(+)-Catechin	ND ± ND	923 ± 35	567 ± 28	1632 ± 100
catechin-glucoside	ND ± ND	3.21 ± 0.36	14.6 ± 2.0	5.41 ± 0.47
(-)-Epicatechin	ND ± ND	45.0 ± 2.3	88.2 ± 16.0	75.6 ± 4.1
(-)-Epigallocatechin	ND ± ND	114 ± 11	ND ± ND	ND ± ND
(−)-Gallocatechin	ND ± ND	1032 ± 160	ND ± ND	ND ± ND
Flavan-3-ols	ND ± ND	2117 ± 208	670 ± 46	1713 ± 104
Eriodictyol	ND ± ND	1.16 ± 0.14	ND ± ND	ND ± ND
Naringenin	ND ± ND	1.71 ± 0.30	0.38 ± 0.03	0.88 ± 0.42
Flavanones	ND ± ND	2.87 ± 0.44	0.38 ± 0.03	0.88 ± 0.42
3,4-Dihydroxybenzoic acid	0.68 ± 0.08	6.62 ± 0.28	5.98 ± 0.33	6.14 ± 1.65
4-hydroxybenzoic acid	ND ± ND	ND ± ND	82.2 ± 7.4	ND ± ND
Gallic acid	ND ± ND	71.4 ± 5.7	ND ± ND	ND ± ND
Vanillic acid-4-β-D-glucoside	3.70 ± 0.59	191 ± 15	86.1 ± 5.1	51.9 ± 4.0
Hydroxybenzoic acids	4.38 ± 0.67	269 ± 21	174 ± 13	58.0 ± 5.6
Caffeic acid	ND ± ND	ND ± ND	12.2 ± 0.1	0.64 ± 0.28
Chlorogenic acid	ND ± ND	18.8 ± 2.3	45.0 ± 9.3	153 ± 29
Ferulic acid (trans)	ND ± ND	288 ± 84	23.9 ± 5.8	95.1 ± 18.5
p-Coumaric acid (trans)	ND ± ND	ND ± ND	19.4 ± 2.0	ND ± ND

Hydroxycinnamic acids	ND ± ND	307 ± 86	101 ± 17	249 ± 48
Hydroxycoumarins	ND ± ND	ND ± ND	ND ± ND	ND ± ND
Genistein	ND ± ND	ND ± ND	0.48 ± 0.12	ND ± ND
Isoflavones	ND ± ND	ND ± ND	0.48 ± 0.12	ND ± ND
Procyanidin B1	ND ± ND	454 ± 57	239 ± 25	952 ± 100
Procyanidin B3	ND ± ND	49.7 ± 5.5	22.1 ± 1.2	180 ± 27
Procyanidin C1	ND ± ND	70.7 ± 8.9	94.7 ± 6.8	616 ± 98
Procyanidins	ND ± ND	574 ± 71	356 ± 33	1748 ± 225
Resveratrol 3-β-mono-D-glucoside (Polydatin)	ND ± ND	0.43 ± 0.11	ND ± ND	0.32 ± 0.05
Stilbenes	ND ± ND	0.43 ± 0.11	ND ± ND	0.32 ± 0.05

Table S5. Concentrations of polyphenols in lentil seed coat extracts (µmol/g dry weight)

Compound	L1			L2			L3			L4		
	Mean	±	SD	Mean	±	SD	Mean	±	SD	Mean	±	SD
Delphinidin 3-O-rhamnoside	ND	±	ND	1.23	±	0.38	ND	±	ND	ND	±	ND
Anthocyanins	ND	±	ND	1.23	±	0.38	ND	±	ND	ND	±	ND
Chalcones	ND	±	ND	ND	±	ND	ND	±	ND	ND	±	ND
Dihydromyricetin	ND	±	ND	4.95	±	0.42	5.15	±	0.57	2.31	±	0.21
Dihydrokaempferol	2.52	±	0.50	0.38	±	0.05	0.39	±	0.05	0.32	±	0.04
Taxifolin (dihydroquercetin)	0.83	±	0.19	2.52	±	0.24	2.81	±	0.12	1.66	±	0.24
Dihydroflavonols	3.35	±	0.69	7.85	±	0.71	8.35	±	0.74	4.29	±	0.49
Apigenin	0.71	±	0.14	1.88	±	0.38	ND	±	ND	0.42	±	0.10
Diosmetin	ND	±	ND	1.21	±	0.20	0.36	±	0.01	ND	±	ND
Luteolin	0.48	±	0.08	264	±	39	39.8	±	8.2	13.6	±	1.6
Luteolin 4'-O-glucoside	0.59	±	0.23	323	±	25	50.1	±	2.2	39.5	±	4.5
Luteolin 7-O-glucoside	ND	±	ND	3.71	±	0.38	0.47	±	0.10	ND	±	ND
Flavones	1.78	±	0.45	594	±	64	90.7	±	10.6	53.5	±	6.2
Kaempferol 3-O-rhamnoside	1.57	±	0.36	4.45	±	0.47	4.13	±	0.49	8.01	±	0.31
Fisetin	0.87	±	0.28	1.03	±	0.32	ND	±	ND	ND	±	ND
Kaempferol 3-O-robinoside-7-O-rhamnoside (robinin)	37.7	±	8.1	21.3	±	2.2	85.8	±	8.0	23.8	±	4.1
Kaempferol 7-O-glucoside	ND	±	ND	0.60	±	0.08	0.31	±	0.04	ND	±	ND
Kaempferol dirutinoside	394	±	83	200	±	8	386	±	49	243	±	28
Myricetin	ND	±	ND	20.1	±	3.4	9.14	±	2.59	6.13	±	0.69
Myricetin 3-O-rhamnoside	ND	±	ND	20.1	±	3.3	8.59	±	1.97	5.89	±	0.66
Quercetin	ND	±	ND	3.13	±	0.75	1.18	±	0.09	0.93	±	0.18
Quercetin 3-O-glucoside (Isoquercetrin)	ND	±	ND	0.29	±	0.09	0.35	±	0.03	0.34	±	0.01

Quercetin 3-O-rhamnoside (Quercitrin)	0.38 ± 0.07	12.5 ± 1.0	10.4 ± 0.8	10.0 ± 0.3
Quercetin 3-O-rutinoside(Rutin)	ND ± ND	0.88 ± 0.07	1.12 ± 0.17	0.80 ± 0.08
Flavonols	435 ± 92	284 ± 20	507 ± 63	299 ± 34
(+)-Catechin	2.03 ± 0.30	195 ± 12	360 ± 24	157 ± 15
catechin-glucoside	141 ± 24	2561 ± 275	6763 ± 1314	2787 ± 369
(-)-Epicatechin	ND ± ND	5.17 ± 0.27	8.73 ± 1.09	3.82 ± 0.97
(-)-Epigallocatechin	1.10 ± 0.20	5.65 ± 0.38	4.83 ± 0.59	2.82 ± 0.27
(−)-Gallocatechin	0.72 ± 0.05	53.4 ± 7.2	314 ± 39	111 ± 6
Flavan-3-ols	145 ± 24	2820 ± 295	7451 ± 1378	3062 ± 391
Eriodictyol	ND ± ND	0.60 ± 0.08	1.12 ± 0.23	0.57 ± 0.08
Naringenin	ND ± ND	0.65 ± 0.10	0.80 ± 0.11	0.91 ± 0.10
Flavanones	ND ± ND	1.25 ± 0.18	1.92 ± 0.34	1.48 ± 0.18
3,4-Dihydroxybenzoic acid	9.09 ± 0.95	16.1 ± 0.6	4.53 ± 0.98	5.91 ± 0.89
Gallic acid	ND ± ND	99.5 ± 10.4	6.75 ± 1.72	4.21 ± 0.50
Vanillic acid 4-β-D-glucoside	587 ± 89	63.2 ± 5.5	293 ± 30	279 ± 16
Vanillin	0.93 ± 0.81	ND ± ND	ND ± ND	ND ± ND
Hydroxybenzoic acids	597 ± 90	179 ± 17	304 ± 33	289 ± 17
Caffeic acid	1.70 ± 0.13	0.63 ± 0.16	0.83 ± 0.35	1.47 ± 0.52
Chlorogenic acid	ND ± ND	ND ± ND	5.48 ± 1.53	3.50 ± 1.00
p-Coumaric acid (trans)	12.5 ± 1.8	14.2 ± 3.8	29.0 ± 6.5	14.7 ± 0.9
Hydroxycinnamic acids	14.2 ± 1.9	14.8 ± 3.9	35.3 ± 8.4	19.7 ± 2.4
Hydroxycoumarins	ND ± ND	ND ± ND	ND ± ND	ND ± ND
Isoflavones	ND ± ND	ND ± ND	ND ± ND	ND ± ND
Procyanidin B1	1.38 ± 0.04	200 ± 21	162 ± 21	186 ± 19
Procyanidin B3	14.6 ± 1.3	1150 ± 91	1401 ± 80	1058 ± 76
Procyanidin C1	6.55 ± 1.05	512 ± 40	606 ± 74	554 ± 33
Procyanidins	22.5 ± 2.3	1862 ± 152	2169 ± 175	1798 ± 128

Resveratrol 3-β-mono-D-glucoside (Polydatin)	0.78 ± 0.04	21.2 ± 1.4	21.8 ± 3.8	16.8 ± 0.3
Stilbenes	0.78 ± 0.04	21.2 ± 1.4	21.8 ± 3.8	16.8 ± 0.3

Table S6. Concentrations of polyphenols in pea seed coat extracts (µmol/g dry weight)

Compound	P1			P2			P3			P4		
	Mean	±	SD	Mean	±	SD	Mean	±	SD	Mean	±	SD
Anthocyanins	ND	±	ND	ND	±	ND	ND	±	ND	ND	±	ND
Chalcones	ND	±	ND	ND	±	ND	ND	±	ND	ND	±	ND
Dihydromyricetin	1.39	±	0.16	18.8	±	3.9	0.38	±	0.03	30.7	±	2.8
Dihydrokaempferol	ND	±	ND	0.53	±	0.17	2.53	±	0.29	0.75	±	0.05
Taxifolin (dihydroquercetin)	4.31	±	1.30	2.26	±	0.50	1.64	±	0.37	9.83	±	0.95
Dihydroflavonols	5.70	±	1.46	21.6	±	4.6	4.55	±	0.69	41.3	±	3.8
Vitexin	ND	±	ND	131	±	14	ND	±	ND	44.5	±	4.0
Luteolin	ND	±	ND	5.98	±	0.72	ND	±	ND	8.45	±	1.14
Luteolin 7-O-glucoside	ND	±	ND	1.62	±	0.21	ND	±	ND	1.23	±	0.13
Luteolin 8'-C-glucoside	ND	±	ND	61.6	±	17.4	ND	±	ND	32.9	±	9.4
Flavones	ND	±	ND	200	±	32	ND	±	ND	87.1	±	14.6
Kaempferol 3-O-rhamnoside	ND	±	ND	3.97	±	0.18	ND	±	ND	5.45	±	0.57
Kaempferol 3-O-rutinoside-4'-glucoside	0.22	±	0.06	ND	±	ND	ND	±	ND	ND	±	ND
Kaempferol 3-O-glucoside	1.47	±	0.18	2.24	±	0.61	6.21	±	1.07	2.77	±	0.59
Kaempferol 7-O-glucoside	ND	±	ND	0.58	±	0.10	ND	±	ND	0.57	±	0.08
Myricetin	ND	±	ND	15.6	±	1.5	ND	±	ND	15.5	±	3.7
Myricetin 3-O-rhamnoside	ND	±	ND	80.3	±	15.3	ND	±	ND	92.4	±	11.0
Quercetin	0.73	±	0.05	0.36	±	0.04	0.94	±	0.56	0.91	±	0.14
Quercetin 3-O-glucoside (Isoquercetrin)	0.33	±	0.01	ND	±	ND	ND	±	ND	0.41	±	0.04
Quercetin 3-O-rhamnoside (Quercitrin)	ND	±	ND	9.16	±	0.49	ND	±	ND	21.0	±	2.0
Flavonols	2.87	±	0.33	112	±	18	7.15	±	1.63	139	±	18
(+)-Catechin	ND	±	ND	1.33	±	0.30	ND	±	ND	1.36	±	0.24
(-)-Epicatechin	ND	±	ND	3.46	±	0.40	ND	±	ND	10.7	±	0.6

(-)-Epigallocatechin	ND ± ND	847 ± 175	ND ± ND	994 ± 169
(−)-Gallocatechin	ND ± ND	179 ± 40	ND ± ND	76.9 ± 12.9
Flavan-3-ols	ND ± ND	1031 ± 216	ND ± ND	1083 ± 183
Eriodictyol	ND ± ND	1.08 ± 0.16	ND ± ND	2.61 ± 0.24
Naringenin	ND ± ND	3.78 ± 0.28	3.06 ± 0.05	3.59 ± 0.40
Flavanones	ND ± ND	4.86 ± 0.44	3.06 ± 0.05	6.20 ± 0.64
3,4-Dihydroxybenzoic acid	75.7 ± 10.7	181 ± 32	17.9 ± 4.6	530 ± 82
4-hydroxybenzoic acid	ND ± ND	255 ± 53	ND ± ND	142 ± 16
Gallic acid	ND ± ND	35.9 ± 7.3	ND ± ND	121 ± 26
Vanillic acid	68.7 ± 20.7	468 ± 98	122 ± 19	104 ± 6
Vanillic acid 4-β-D-glucoside	16.6 ± 1.1	132 ± 15	9.01 ± 1.52	76.5 ± 23.7
Vanillin	9.17 ± 2.44	ND ± ND	ND ± ND	ND ± ND
Hydroxybenzoic acids	170 ± 35	1072 ± 206	149 ± 26	974 ± 154
Caffeic acid	2.42 ± 0.32	1.79 ± 0.09	ND ± ND	8.22 ± 1.42
Ferulic acid (trans)	22.6 ± 0.9	85.9 ± 6.6	8.12 ± 2.43	6.95 ± 0.22
Hydroxycinnamic acids	25.0 ± 1.2	87.7 ± 6.6	8.12 ± 2.43	15.2 ± 1.6
Hydroxycoumarins	ND ± ND	ND ± ND	ND ± ND	ND ± ND
Genistein	ND ± ND	ND ± ND	ND ± ND	1.10 ± 0.12
Prunetin	2.23 ± 0.81	6.18 ± 1.16	19.7 ± 2.6	ND ± ND
Isoflavones	2.23 ± 0.81	6.18 ± 1.16	19.7 ± 2.6	1.10 ± 0.12
Procyanidins	ND ± ND	ND ± ND	ND ± ND	ND ± ND
Resveratrol 3-β-mono-D-glucoside (Polydatin)	ND ± ND	2.27 ± 0.21	ND ± ND	9.75 ± 0.99
Stilbenes	ND ± ND	2.27 ± 0.21	ND ± ND	9.75 ± 0.99

Table S7. Concentrations of polyphenols in chickpea seed coat extracts (μmol/g dry weight)

Compound	C1			C2			C3			C4		
	Mean	±	SD	Mean	±	SD	Mean	±	SD	Mean	±	SD
Anthocyanins	ND	±	ND	ND	±	ND	ND	±	ND	ND	±	ND
Chalcones	ND	±	ND	ND	±	ND	ND	±	ND	ND	±	ND
Dihydromyricetin	ND	±	ND	8.74	±	1.88	7.41	±	0.52	7.40	±	1.30
Dihydrokaempferol	ND	±	ND	ND	±	ND	0.75	±	0.08	ND	±	ND
Taxifolin (dihydroquercetin)	ND	±	ND	0.67	±	0.08	1.49	±	0.19	0.68	±	0.16
Dihydroflavonols	ND	±	ND	9.41	±	1.96	9.65	±	0.79	8.08	±	1.46
Apigenin	0.81	±	0.22	ND	±	ND	ND	±	ND	ND	±	ND
Luteolin	0.54	±	0.24	0.59	±	0.13	0.87	±	0.17	0.90	±	0.48
Flavones	1.35	±	0.46	0.59	±	0.13	0.87	±	0.17	0.90	±	0.48
Kaempferol 3-O-rhamnoside	ND	±	ND	0.26	±	0.06	0.76	±	0.13	0.34	±	0.09
Isorhamnetin	0.90	±	0.10	3.22	±	0.41	4.93	±	0.98	5.89	±	0.88
Kaempferol	1.08	±	0.22	1.35	±	0.17	2.76	±	0.47	4.61	±	1.36
Kaempferol 3-O-D-galactoside	ND	±	ND	0.30	±	0.09	1.10	±	0.08	0.61	±	0.11
Kaempferol 3-O-glucoside	65.9	±	19.5	12.9	±	2.2	40.6	±	5.5	24.2	±	9.0
Kaempferol 3-O-rutinoside	0.59	±	0.04	68.3	±	18.4	117	±	6	48.0	±	8.4
Kaempferol di-rutinoside	0.19	±	0.01	ND	±	ND	ND	±	ND	ND	±	ND
Myricetin	ND	±	ND	34.5	±	8.5	28.7	±	5.3	32.8	±	8.4
Myricetin 3-O-rhamnoside	ND	±	ND	2.39	±	0.44	3.81	±	0.68	1.36	±	0.24
Quercetin	0.89	±	0.40	4.59	±	0.78	10.5	±	1.7	10.0	±	1.8
Quercetin 3-O-galactoside	1.29	±	0.21	1.43	±	0.31	3.34	±	0.65	2.19	±	0.34
Quercetin 3-O-glucoside (Isoquercetrin)	3.66	±	0.49	1.60	±	0.54	4.32	±	0.41	1.82	±	0.18
Quercetin 3-O-rhamnoside (Quercitrin)	ND	±	ND	0.49	±	0.08	1.30	±	0.21	0.53	±	0.05

Quercetin 3-O-rutinoside(Rutin)	1.27 ± 0.18	159 ± 45	281 ± 20	149 ± 34
Flavonols	75.8 ± 21.2	290 ± 77	500 ± 42	281 ± 64
(-)-Epigallocatechin	ND ± ND	4.03 ± 0.24	9.22 ± 0.69	6.76 ± 0.91
(−)-Gallocatechin	ND ± ND	63.1 ± 9.9	113 ± 2	105 ± 15
Flavan-3-ols	ND ± ND	67.1 ± 10.2	122 ± 3	112 ± 16
Naringenin	0.41 ± 0.06	1.22 ± 0.21	0.53 ± 0.09	0.48 ± 0.12
Flavanones	0.41 ± 0.06	1.22 ± 0.21	0.53 ± 0.09	0.48 ± 0.12
3,4-Dihydroxybenzoic acid	2.05 ± 0.50	3.78 ± 0.10	3.83 ± 0.47	2.45 ± 0.15
4-hydroxybenzoic acid	216 ± 41	115 ± 3	ND ± ND	ND ± ND
Gallic acid	ND ± ND	336 ± 39	163 ± 5	150 ± 21
Vanillic acid 4-β-D-glucoside	10.6 ± 1.9	4.79 ± 0.67	13.5 ± 1.1	9.93 ± 1.21
Vanillin	7.62 ± 1.11	ND ± ND	ND ± ND	ND ± ND
Hydroxybenzoic acids	236 ± 44	460 ± 42	180 ± 7	162 ± 23
Ferulic acid (trans)	ND ± ND	15.7 ± 3.5	ND ± ND	41.0 ± 7.5
Hydroxycinnamic acids	ND ± ND	15.7 ± 3.5	ND ± ND	41.0 ± 7.5
Hydroxycoumarins	ND ± ND	ND ± ND	ND ± ND	ND ± ND
Prunetin	2.30 ± 0.10	0.49 ± 0.13	ND ± ND	ND ± ND
Isoflavones	2.30 ± 0.10	0.49 ± 0.13	ND ± ND	ND ± ND
Procyanidins	ND ± ND	ND ± ND	ND ± ND	ND ± ND
Stilbenes	ND ± ND	ND ± ND	ND ± ND	ND ± ND

Table S8. Concentrations of polyphenols in faba bean seed coat extracts (μmol/g dry weight)

Compound	F1			F2			F3			F4		
	Mean	±	SD	Mean	±	SD	Mean	±	SD	Mean	±	SD
Anthocyanins	ND	±	ND	ND	±	ND	ND	±	ND	ND	±	ND
Chalcones	ND	±	ND	ND	±	ND	ND	±	ND	ND	±	ND
Dihydromyricetin	1.66	±	0.67	4.68	±	1.11	9.96	±	1.67	10.1	±	1.1
Taxifolin (dihydroquercetin)	1.55	±	0.46	1.64	±	0.28	2.21	±	0.12	2.59	±	0.39
Dihydroflavaonols	3.21	±	1.13	6.32	±	1.39	12.2	±	1.8	12.7	±	1.5
Vitexin	ND	±	ND	6.99	±	1.38	0.62	±	0.09	1.39	±	0.09
Luteolin	0.90	±	0.38	0.77	±	0.14	0.41	±	0.07	1.15	±	0.15
Luteolin 4'-O-glucoside	ND	±	ND	0.30	±	0.04	0.43	±	0.07	0.35	±	0.07
Luteolin 8'-C-glucoside	ND	±	ND	0.77	±	0.20	0.24	±	0.01	0.30	±	0.07
Flavones	0.90	±	0.38	8.83	±	1.76	1.70	±	0.24	3.19	±	0.38
Isorhamnetin	2.85	±	0.38	2.19	±	0.38	0.37	±	0.34	1.01	±	0.11
Kaempferol 3-O-robinoside-7-O-rhamnoside (robinin)	3.33	±	0.48	0.36	±	0.02	3.25	±	0.63	3.05	±	0.28
Kaempferol 3-O-D-galactoside	0.51	±	0.43	ND	±	ND	ND	±	ND	ND	±	ND
Kaempferol 3-O-glucoside	2.55	±	0.39	3.93	±	0.32	ND	±	ND	ND	±	ND
Kaempferol 3-O-rutinoside	0.22	±	0.02	6.36	±	0.05	ND	±	ND	0.58	±	0.03
Myricetin	1.20	±	0.46	13.6	±	2.0	5.72	±	0.93	9.23	±	2.16
Myricetin 3-O-rhamnoside	ND	±	ND	5.71	±	1.32	8.36	±	1.93	25.9	±	4.9
Quercetin	5.59	±	1.14	6.01	±	1.86	1.25	±	0.25	5.01	±	0.73
Quercetin 3-O-galactoside	0.35	±	0.15	1.05	±	0.08	0.46	±	0.08	0.66	±	0.13
Quercetin 3-O-glucoside (Isoquercetrin)	1.89	±	0.34	1.12	±	0.01	0.37	±	0.06	0.87	±	0.05
Quercetin 3-O-rhamnoside (Quercitrin)	ND	±	ND	1.46	±	0.20	1.29	±	0.09	3.90	±	0.34
Quercetin 3-O-rutinoside(Rutin)	1.37	±	0.13	0.18	±	0.02	3.42	±	0.50	2.28	±	0.40
Flavonols	19.9	±	3.9	42.0	±	6.3	24.5	±	4.8	52.5	±	9.1

(+)-Catechin	ND ± ND	48.1 ± 5.9	61.2 ± 3.2	142 ± 30
catechin-glucoside	ND ± ND	1.38 ± 0.31	1.45 ± 0.16	7.37 ± 0.40
(-)-Epicatechin	ND ± ND	76.4 ± 6.1	1587 ± 158	137 ± 10
(-)-Epigallocatechin	ND ± ND	82.2 ± 6.1	1078 ± 185	75.3 ± 8.9
(−)-Gallocatechin	ND ± ND	33.5 ± 1.6	30.3 ± 2.8	71.9 ± 6.4
Flavan-3-ols	ND ± ND	242 ± 20	2758 ± 349	434 ± 56
Naringenin	ND ± ND	0.55 ± 0.11	0.47 ± 0.03	0.68 ± 0.08
Flavanones	ND ± ND	0.55 ± 0.11	0.47 ± 0.03	0.68 ± 0.08
3,4-Dihydroxybenzoic acid	5.98 ± 0.22	13.7 ± 0.8	14.2 ± 1.9	28.8 ± 2.5
Gallic acid	0.95 ± 0.03	42.4 ± 5.3	30.7 ± 3.7	53.5 ± 4.1
Vanillic acid 4-β-D-glucoside	4.88 ± 0.62	14.9 ± 2.2	24.2 ± 1.0	20.2 ± 2.4
Hydroxybenzoic acids	11.8 ± 0.9	71.0 ± 8.3	69.1 ± 6.6	103 ± 9
Caffeic acid	14.9 ± 2.1	0.50 ± 0.07	1.15 ± 0.11	3.38 ± 0.43
Chlorogenic acid	ND ± ND	9.10 ± 1.00	170 ± 29	74.5 ± 10.6
Hydroxycinnamic acids	14.9 ± 2.1	9.60 ± 1.07	171 ± 29	77.9 ± 11.0
Hydroxycoumarins	ND ± ND	ND ± ND	ND ± ND	ND ± ND
Isoflavones	ND ± ND	ND ± ND	ND ± ND	ND ± ND
Procyanidin B1	ND ± ND	38.1 ± 4.1	389 ± 35	228 ± 21
Procyanidin B3	ND ± ND	36.4 ± 4.4	488 ± 72	298 ± 16
Procyanidin C1	ND ± ND	11.0 ± 2.5	142 ± 4	89.0 ± 6.4
Procyanidins	ND ± ND	85.5 ± 11.0	1019.00 ± 110.00	615.00 ± 43.40
Resveratrol 3-β-mono-D-glucoside (Polydatin)	ND ± ND	14.2 ± 1.7	8.13 ± 1.40	13.3 ± 0.3
Stilbenes	ND ± ND	14.2 ± 1.7	8.13 ± 1.40	13.3 ± 0.3

Table S9. Estimated amounts (µmol/g) of major polyphenols detected in common bean seed coats by the untargeted method and not quantified in Table 4. Vanillic acid 4-β-D-glucoside was used to estimate the amounts of phenolic acids, whereas kaempferol 3-O-rutinoside, quercetin 3-O-rhamnoside, myricetin 3-O-rhamnoside, luteolin 4'-O-glucoside and delphinidin 3-β-D-glucoside were used to estimate the amounts of kaempferol, quercetin and myricetin compounds, flavones and anthocyanins, respectively. Flavan-3-ols were estimated as catechin equivalents, while procyanidins, prodelphinidins and propelargonidins were estimated as procyanidin B1 equivalent.

Name	Formula	Molecular Weight	RT [min]	B1	B2	B3	B4
Dihydroxybenzoic acid	C7 H6 O4	154.02667	10.99	ND	ND	ND	ND
Phenolic acid derivative	C11 H12 O5	224.06867	10.99	1	ND	ND	3
Phenolic acid derivative	C11 H12 O6	240.06358	8.54	72	215	558	221
Phenolic acid derivative	C12 H16 O5	240.10005	9.19	7	5	3	5
Phenolic acid derivative	C13 H20 O4	240.13636	12.97	25	32	6	21
Phenolic acid derivative	C12 H18 O5	242.1157	5.86	1	2	5	3
Phenolic acid derivative	C12 H18 O6	258.11044	5.42	ND	ND	ND	ND
Phenolic acid derivative	C12 H18 O6	258.11047	6.24	ND	ND	ND	ND
Phenolic acid derivative	C12 H14 O7	270.07392	9.62	ND	ND	ND	ND
Phenolic acid derivative	C14 H24 O5	272.16243	10.59	257	25	191	ND
Phenolic acid derivative	C14 H24 O5	272.1625	9.89	365	61	195	ND
Hydroxybenzoic acid hexoside	C13 H16 O8	300.08462	6.40	ND	2	11	2
Hydroxybenzoic acid hexoside	C13 H16 O8	300.08472	3.94	2	72	71	76
Dihydroxybenzoic acid hexoside	C13 H16 O9	316.07957	8.48	ND	1	15	2
Dihydroxybenzoic acid hexoside	C13 H16 O9	316.07964	8.97	ND	16	5	8
Trihydroxybenzoic acid hexoside	C13 H16 O10	332.07459	13.38	ND	3	37	4
Phenolic acid deoxyhexoside	C15 H16 O10	356.07484	9.66	ND	17	222	1
Phenolic acid derivative	C15 H18 N O9 P	387.07262	12.08	ND	ND	ND	ND
Hydroxybenzoic acid derivative	C16 H20 O11	388.10104	9.22	2	2	ND	1

Phenolic acid derivative	C18 H28 O9	388.17373	7.18	26	42	19	92
Phenolic acid derivative	C18 H28 O10	404.16868	8.59	ND	ND	ND	ND
Phenolic acid derivative	C18 H28 O10	404.1687	11.13	ND	ND	ND	ND
5-O-[B-apiosyl-(1-2)-O-B-xylopyranosyl]gentisic acid	C17 H22 O12	418.11142	10.98	ND	ND	18	15
Caffeic acid malonyl glucoside	C16 H28 O13	428.15318	2.24	ND	ND	ND	ND
Hydroxybenzoic acid hexoside pentoside	C18 H24 O12	432.12679	5.74	25	61	2	96
Dihydroxybenzoic acid pentoside hexoside	C18 H24 O13	448.12215	9.43	ND	12	5	10
Phenolic acid derivative	C20 H28 O14	492.14852	7.91	ND	25	5	1
Phenolic acid derivative	C21 H32 O13	492.18471	12.43	4	9	ND	8
Phenolic acid dihexoside derivative	C28 H34 O18	658.17597	6.47	ND	210	ND	ND
Phenolic acid dihexoside derivative	C29 H36 O18	672.19118	7.75	ND	232	ND	ND
Phenolic acids				787	1044	1368	569
Kaempferol acetyl hexoside	C23 H22 O12	490.11134	16.39	2	832	4761	1975
Kaempferol dihexoside	C27 H30 O16	610.15424	12.47	ND	1	1884	45
Kaempferol malonyl hexoside	C24 H22 O14	534.10118	16.40	5	2403	19865	7070
Kaempferol 3-O-sambioside (leucoside)	C26 H28 O15	580.1433	13.41	10	127	9239	1510
Kaempferol pentoside-hexoside-deoxyhexoside	C32 H38 O19	726.20143	12.95	ND	ND	ND	ND
Myricetin derivative	C34 H42 O22	802.21764	11.20	ND	ND	ND	ND
Myricetin 3-O-glucoside	C21 H20 O13	480.09095	12.36	ND	5539	154	2
Myricetin hexoside deoxyhexoside	C27 H30 O17	626.14864	12.19	ND	ND	4	ND
Myricetin hexoside deoxyhexoside	C27 H30 O17	626.14869	12.11	ND	17	ND	ND
Myricetin hexoside dideoxyhexoside	C33 H40 O21	772.20664	12.29	ND	ND	ND	ND
Myricetin pentoside-hexoside-deoxyhexoside	C32 H38 O21	758.19094	11.32	ND	1	1	ND
Myricetin pentoside-hexoside-deoxyhexoside	C32 H38 O21	758.19098	11.55	ND	8	ND	ND
Quercetin deoxyhexoside	C21 H20 O11	448.10092	16.75	ND	ND	2	ND

Quercetin hexoside derivative	C24 H26 O13	522.13792	10.21	ND	78	ND	ND
Quercetin hexoside derivative	C24 H26 O13	522.13791	9.48	ND	100	ND	ND
Quercetin pentoside	C17 H22 O13	434.10639	14.31	ND	ND	ND	ND
Quercetin pentoside-hexoside-deoxyhexoside	C32 H38 O20	742.19611	12.15	ND	ND	ND	ND
Quercetin pentoside-hexoside-deoxyhexoside	C32 H38 O20	742.19625	12.24	ND	ND	ND	ND
3,5-Dihydroxy-2-(4-hydroxyphenyl)-4-oxo-3,4-dihydro-2H-chromen-7-yl hexopyranoside	C21 H22 O11	450.11658	9.89	ND	32	877	133
Flavonols				17	9138	36787	10735
Tricetin hexoside	C21 H20 O12	464.09605	16.75	ND	5	ND	ND
Tricetin hexoside	C21 H20 O12	464.09606	15.16	ND	ND	ND	ND
Flavones				0	5	0	0
Afzelechin	C15 H14 O5	274.0845	10.29	ND	ND	167	11
Epi-afzelechin	C15 H14 O5	274.08451	11.15	ND	1	174	55
(Epi)afzelechin hexoside	C21 H24 O10	436.13738	7.70	ND	3	262	308
(Epi)catechin hexoside	C21 H24 O11	452.13217	6.59	ND	484	386	1714
(Epi)catechin hexoside	C21 H24 O11	452.13245	9.34	ND	1	5	2
(Epi)gallocatechin hexoside	C21 H24 O12	468.12707	4.85	ND	261	ND	ND
(Epi)gallocatechin hexoside	C21 H24 O12	468.12722	7.88	ND	ND	ND	ND
Flavan-3-ols				0	750	994	2090
AA	C30 H26 O10	546.15358	11.42	ND	ND	302	80
AA	C30 H26 O10	546.1536	10.35	ND	ND	239	39
AC	C30 H26 O11	562.148	9.14	ND	98	792	1447
AAC	C45 H38 O16	834.2176	10.94	ND	1	180	130
ACC	C45 H38 O17	850.21255	10.25	ND	7	117	394
ACC	C45 H38 O17	850.21255	9.91	ND	9	61	222
ACCC	C60 H50 O23	1138.27613	10.61	ND	3	71	360
ACCCC	C75 H62 O29	1426.3409	11.45	ND	4	75	446
Propelargonidins				0	122	1837	3118

CC	C30 H26 O12	578.14271	9.83	ND	3	4	2
CC	C30 H26 O12	578.14298	10.40	ND	4	3	40
CC	C30 H26 O12	578.14315	11.61	ND	71	76	520
CCC	C45 H38 O18	866.20698	10.87	ND	8	25	84
CCC	C45 H38 O18	866.207	6.03	ND	56	20	464
CCC	C45 H38 O18	866.20703	9.25	ND	94	142	1147
CCC	C45 H38 O18	866.20715	9.73	ND	25	38	495
CCCC	C60 H50 O24	1154.27068	9.84	ND	7	8	360
CCCC	C60 H50 O24	1154.2707	9.97	ND	12	32	276
CCCC	C60 H50 O24	1154.27127	11.69	ND	11	83	250
CCCCC	C75 H62 O30	1442.33558	10.99	ND	9	40	573
CCCCC	C75 H62 O30	1442.33568	10.45	ND	13	12	271
CCCCCC	C90 H74 O36	1730.39828	11.68	ND	4	18	209
CCCCCCC	C105 H86 O42	2018.46508	12.00	ND	ND	4	194
Procyanidins				0	317	505	4885
GGCCC	C75 H62 O32	1474.32534	9.09	ND	74	ND	ND
GGGGC	C75 H62 O34	1506.31495	7.97	ND	288	ND	ND
GC	C30 H26 O13	594.13772	8.67	ND	12	ND	ND
GC	C30 H26 O13	594.13794	7.53	ND	153	ND	ND
GC	C30 H26 O13	594.13794	6.68	ND	259	ND	ND
GC	C30 H26 O13	594.13794	7.22	ND	24	ND	ND
GC	C30 H26 O13	594.13798	6.32	ND	613	ND	ND
GC	C30 H26 O13	594.13803	9.01	ND	9	ND	2
GCC	C45 H38 O19	882.20148	9.54	ND	9	ND	ND
GCC	C45 H38 O19	882.20187	8.24	ND	381	ND	2
GCC	C45 H38 O19	882.2021	8.36	ND	53	ND	ND
GCC	C45 H38 O19	882.20213	5.17	ND	75	ND	ND
GGC	C45 H38 O20	898.19605	4.78	ND	88	ND	ND
GGC	C45 H38 O20	898.19622	7.43	ND	402	ND	ND
GGC	C45 H38 O20	898.19641	7.34	ND	66	ND	ND

GGC	C45 H38 O20	898.19674	9.98	ND	26	ND	ND
GCCC	C60 H50 O25	1170.26587	9.38	ND	34	ND	ND
GGCC	C60 H50 O26	1186.26057	9.66	ND	17	ND	ND
GGCC	C60 H50 O26	1186.26075	8.56	ND	6	ND	ND
GGGC	C60 H50 O27	1202.25548	7.85	ND	21	ND	ND
GCCCC	C75 H62 O31	1458.33039	10.23	ND	6	ND	2
GGCCC	C75 H62 O32	1474.32422	10.53	ND	13	ND	ND
GGCCC	C75 H62 O32	1474.32441	9.35	ND	58	ND	ND
GGGCC	C75 H62 O33	1490.31944	8.78	ND	23	ND	ND
GGGCC	C75 H62 O33	1490.31961	9.02	ND	192	ND	ND
GGGGC	C75 H62 O34	1506.31474	8.12	ND	3	ND	ND
GGGGC	C75 H62 O34	1506.31576	8.30	ND	66	ND	ND
GGGCCC	C90 H74 O39	1778.38314	9.71	ND	40	ND	2
GGGGCC	C90 H74 O40	1794.37975	9.19	ND	128	ND	ND
GGGGGC	C90 H74 O41	1810.37349	8.47	ND	93	ND	ND
GGGCCCC	C105 H86 O45	2066.4502	10.55	ND	29	ND	ND
GGGGCCC	C105 H86 O46	2082.44459	9.92	ND	9	ND	ND
“G-C”-prodelphinidins				0	3270	0	8
GG	C30 H26 O14	610.13302	6.17	ND	13	ND	ND
GG	C30 H26 O14	610.13303	8.24	ND	7	ND	1
GG	C30 H26 O14	610.13304	4.82	ND	614	ND	ND
GG	C30 H26 O14	610.13321	6.48	ND	92	ND	ND
GG-deoxyhexoside	C33 H40 O20	756.21216	13.14	ND	ND	ND	ND
GGG	C45 H38 O21	914.1916	7.72	ND	27	ND	ND
GGG	C45 H38 O21	914.19169	6.63	ND	177	ND	ND
GGG	C45 H38 O21	914.19184	4.37	ND	60	ND	ND
GGG	C45 H38 O21	914.19192	7.82	ND	32	ND	ND
GGGG	C60 H50 O28	1218.2508	7.33	ND	5	ND	ND
GGGG	C60 H50 O28	1218.25088	9.58	ND	1	ND	ND
GGGGG	C75 H62 O35	1522.3086	10.05	ND	5	ND	ND

GGGGG	C75 H62 O35	1522.30948	7.66	ND	3	ND	ND
GGGGGG	C90 H74 O42	1826.36757	8.90	ND	1	ND	ND
GGGGGGG	C105 H86 O49	2130.43022	9.17	ND	7	ND	ND
“G”-prodelphinidins				0	1044	0	1
Delphinidin 3-O-(2-O-β-d-Glucopyranosyl-α-l-arabinopyranoside)	C26 H28 O16	596.13879	6.68	ND	ND	ND	ND
Anthocyanins				0	0	0	0

Table S10. Estimated amounts (μmol/g) of major polyphenols detected in lentil seed coats by the untargeted method and not quantified in Table 5. Vanillic acid 4-β-D-glucoside was used to estimate the amounts of phenolic acids, whereas kaempferol 3-O-rutinoside, quercetin 3-O-rhamnoside, myricetin 3-O-rhamnoside, luteolin 4'-O-glucoside and delphinidin 3-β-D-glucoside were used to estimate the amounts of kaempferol, quercetin and myricetin compounds, flavones and anthocyanins, respectively. Flavan-3-ols were estimated as catechin equivalents, while procyanidins, prodelphinidins and propelargonidins were estimated as procyanidin B1 equivalent.

Name	Formula	Molecular Weight	RT [min]	L1	L2	L3	L4
Dihydroxybenzoic acid	C7 H6 O4	154.02667	10.99	ND	ND	ND	ND
Phenolic acid derivative	C11 H12 O5	224.06867	10.99	ND	ND	ND	ND
Phenolic acid derivative	C11 H12 O6	240.06358	8.54	ND	ND	ND	ND
Phenolic acid derivative	C12 H16 O5	240.10005	9.19	7	4	2	3
Phenolic acid derivative	C13 H20 O4	240.13636	12.97	904	274	183	587
Phenolic acid derivative	C12 H18 O5	242.1157	5.86	6	3	3	4
Phenolic acid derivative	C12 H18 O6	258.11044	5.42	ND	ND	ND	ND
Phenolic acid derivative	C12 H18 O6	258.11047	6.24	ND	ND	ND	ND
Phenolic acid derivative	C12 H14 O7	270.07392	9.62	ND	ND	ND	ND
Phenolic acid derivative	C14 H24 O5	272.16243	10.59	3	ND	ND	ND
Phenolic acid derivative	C14 H24 O5	272.1625	9.89	6	ND	2	ND
Hydroxybenzoic acid hexoside	C13 H16 O8	300.08462	6.40	786	254	110	201
Hydroxybenzoic acid hexoside	C13 H16 O8	300.08472	3.94	868	99	266	238
Dihydroxybenzoic acid hexoside	C13 H16 O9	316.07957	8.48	3	2	2	2
Dihydroxybenzoic acid hexoside	C13 H16 O9	316.07964	8.97	53	63	65	95
Trihydroxybenzoic acid hexoside	C13 H16 O10	332.07459	13.38	2	5	12	6
Phenolic acid deoxyhexoside	C15 H16 O10	356.07484	9.66	ND	ND	ND	ND
Phenolic acid derivative	C15 H18 N O9 P	387.07262	12.08	2	ND	ND	ND
Hydroxybenzoic acid derivative	C16 H20 O11	388.10104	9.22	209	20	43	20

Phenolic acid derivative	C18 H28 O9	388.17373	7.18	49	20	24	30
Phenolic acid derivative	C18 H28 O10	404.16868	8.59	6	ND	ND	ND
Phenolic acid derivative	C18 H28 O10	404.1687	11.13	6	1	2	1
5-O-[B-apiosyl-(1-2)-O-B-xylopyranosyl]gentisic acid	C17 H22 O12	418.11142	10.98	2191	587	994	1027
Caffeic acid malonyl glucoside	C16 H28 O13	428.15318	2.24	2	ND	ND	ND
Hydroxybenzoic acid hexoside pentoside	C18 H24 O12	432.12679	5.74	7	3	9	2
Dihydroxybenzoic acid pentoside hexoside	C18 H24 O13	448.12215	9.43	454	187	256	276
Phenolic acid derivative	C20 H28 O14	492.14852	7.91	3	297	140	105
Phenolic acid derivative	C21 H32 O13	492.18471	12.43	1144	687	864	806
Phenolic acid dihexoside derivative	C28 H34 O18	658.17597	6.47	ND	ND	ND	ND
Phenolic acid dihexoside derivative	C29 H36 O18	672.19118	7.75	ND	ND	ND	ND
Phenolic acids				6711	2506	2977	3403
Kaempferol acetyl hexoside	C23 H22 O12	490.11134	16.39	ND	ND	ND	ND
Kaempferol dihexoside	C27 H30 O16	610.15424	12.47	3	12	12	11
Kaempferol malonyl hexoside	C24 H22 O14	534.10118	16.40	ND	ND	ND	ND
Kaempferol 3-O-sambioside (leucoside)	C26 H28 O15	580.1433	13.41	ND	1	ND	ND
Kaempferol pentoside-hexoside-deoxyhexoside	C32 H38 O19	726.20143	12.95	ND	ND	ND	ND
Myricetin derivative	C34 H42 O22	802.21764	11.20	ND	ND	ND	ND
Myricetin 3-O-glucoside	C21 H20 O13	480.09095	12.36	ND	9	7	6
Myricetin hexoside deoxyhexoside	C27 H30 O17	626.14864	12.19	ND	2	12	3
Myricetin hexoside deoxyhexoside	C27 H30 O17	626.14869	12.11	ND	5	19	4
Myricetin hexoside dideoxyhexoside	C33 H40 O21	772.20664	12.29	ND	ND	ND	ND
Myricetin pentoside-hexoside-deoxyhexoside	C32 H38 O21	758.19094	11.32	ND	ND	ND	ND
Myricetin pentoside-hexoside-deoxyhexoside	C32 H38 O21	758.19098	11.55	ND	ND	ND	ND
Quercetin deoxyhexoside	C21 H20 O11	448.10092	16.75	ND	28	ND	2

Quercetin hexoside derivative	C24 H26 O13	522.13792	10.21	ND	ND	ND	ND
Quercetin hexoside derivative	C24 H26 O13	522.13791	9.48	ND	ND	ND	ND
Quercetin pentoside	C17 H22 O13	434.10639	14.31	27	64	166	111
Quercetin pentoside-hexoside-deoxyhexoside	C32 H38 O20	742.19611	12.15	ND	ND	ND	ND
Quercetin pentoside-hexoside-deoxyhexoside	C32 H38 O20	742.19625	12.24	ND	ND	ND	ND
3,5-Dihydroxy-2-(4-hydroxyphenyl)-4-oxo-3,4-dihydro-2H-chromen-7-yl hexopyranoside	C21 H22 O11	450.11658	9.89	2	2	6	1
Flavonols				32	123	222	138
Tricetin hexoside	C21 H20 O12	464.09605	16.75	ND	217	4	5
Tricetin hexoside	C21 H20 O12	464.09606	15.16	ND	440	22	17
Flavones				0	657	26	22
Afzelechin	C15 H14 O5	274.0845	10.29	ND	ND	ND	ND
Epi-afzelechin	C15 H14 O5	274.08451	11.15	ND	ND	ND	ND
(Epi)afzelechin hexoside	C21 H24 O10	436.13738	7.70	ND	ND	ND	ND
(Epi)catechin hexoside	C21 H24 O11	452.13217	6.59	ND	2	11	3
(Epi)catechin hexoside	C21 H24 O11	452.13245	9.34	ND	ND	ND	ND
(Epi)gallocatechin hexoside	C21 H24 O12	468.12707	4.85	ND	ND	2	ND
(Epi)gallocatechin hexoside	C21 H24 O12	468.12722	7.88	ND	8	ND	ND
Flavan-3-ols				0	10	13	3
AA	C30 H26 O10	546.15358	11.42	ND	ND	ND	ND
AA	C30 H26 O10	546.1536	10.35	ND	ND	ND	ND
AC	C30 H26 O11	562.148	9.14	ND	2	2	1
AAC	C45 H38 O16	834.2176	10.94	ND	ND	ND	ND
ACC	C45 H38 O17	850.21255	10.25	ND	ND	ND	ND
ACC	C45 H38 O17	850.21255	9.91	ND	ND	ND	ND
ACCC	C60 H50 O23	1138.27613	10.61	ND	ND	ND	ND
ACCCC	C75 H62 O29	1426.3409	11.45	ND	ND	ND	ND
Propelargonidins				ND	2	2	1

CC	C30 H26 O12	578.14271	9.83	ND	7	7	3
CC	C30 H26 O12	578.14298	10.40	9	242	325	274
CC	C30 H26 O12	578.14315	11.61	ND	24	16	8
CCC	C45 H38 O18	866.20698	10.87	3	166	206	178
CCC	C45 H38 O18	866.207	6.03	1	212	179	165
CCC	C45 H38 O18	866.20703	9.25	16	552	537	451
CCC	C45 H38 O18	866.20715	9.73	ND	10	7	5
CCCC	C60 H50 O24	1154.27068	9.84	ND	5	3	2
CCCC	C60 H50 O24	1154.2707	9.97	ND	6	1	1
CCCC	C60 H50 O24	1154.27127	11.69	7	92	118	86
CCCCC	C75 H62 O30	1442.33558	10.99	ND	124	179	145
CCCCC	C75 H62 O30	1442.33568	10.45	ND	2	1	1
CCCCCC	C90 H74 O36	1730.39828	11.68	ND	5	9	9
CCCCCCC	C105 H86 O42	2018.46508	12.00	ND	ND	ND	ND
Procyanidins				36	1447	1588	1328
GGCCC	C75 H62 O32	1474.32534	9.09	ND	17	10	23
GGGGC	C75 H62 O34	1506.31495	7.97	ND	67	94	231
GC	C30 H26 O13	594.13772	8.67	8	300	386	334
GC	C30 H26 O13	594.13794	7.53	52	2234	2497	2240
GC	C30 H26 O13	594.13794	6.68	ND	7	29	20
GC	C30 H26 O13	594.13794	7.22	ND	3	ND	3
GC	C30 H26 O13	594.13798	6.32	ND	100	197	152
GC	C30 H26 O13	594.13803	9.01	ND	19	17	26
GCC	C45 H38 O19	882.20148	9.54	ND	186	227	202
GCC	C45 H38 O19	882.20187	8.24	26	1316	1544	1345
GCC	C45 H38 O19	882.2021	8.36	26	1316	16	14
GCC	C45 H38 O19	882.20213	5.17	ND	48	62	61
GGC	C45 H38 O20	898.19605	4.78	ND	48	96	110
GGC	C45 H38 O20	898.19622	7.43	17	839	10	9
GGC	C45 H38 O20	898.19641	7.34	ND	834	889	898

GGC	C45 H38 O20	898.19674	9.98	2	135	155	155
GCCC	C60 H50 O25	1170.26587	9.38	10	252	273	234
GGCC	C60 H50 O26	1186.26057	9.66	5	186	222	217
GGCC	C60 H50 O26	1186.26075	8.56	11	323	9	64
GGGC	C60 H50 O27	1202.25548	7.85	11	590	715	813
GCCCC	C75 H62 O31	1458.33039	10.23	9	261	329	286
GGCCC	C75 H62 O32	1474.32422	10.53	2	142	152	193
GGCCC	C75 H62 O32	1474.32441	9.35	10	344	395	386
GGGCC	C75 H62 O33	1490.31944	8.78	8	320	381	426
GGGCC	C75 H62 O33	1490.31961	9.02	2	148	235	216
GGGGC	C75 H62 O34	1506.31474	8.12	5	220	111	199
GGGGC	C75 H62 O34	1506.31576	8.30	ND	193	6	17
GGGCCC	C90 H74 O39	1778.38314	9.71	ND	281	406	427
GGGGCC	C90 H74 O40	1794.37975	9.19	4	332	573	652
GGGGGC	C90 H74 O41	1810.37349	8.47	ND	119	202	267
GGGCCCC	C105 H86 O45	2066.4502	10.55	ND	130	165	210
GGGGCCC	C105 H86 O46	2082.44459	9.92	ND	171	265	292
“G-C”-prodelphinidins				208	11481	10668	10722
GG	C30 H26 O14	610.13302	6.17	ND	ND	ND	ND
GG	C30 H26 O14	610.13303	8.24	ND	3	2	2
GG	C30 H26 O14	610.13304	4.82	ND	24	80	63
GG	C30 H26 O14	610.13321	6.48	3	247	556	498
GG-deoxyhexoside	C33 H40 O20	756.21216	13.14	ND	ND	ND	ND
GGG	C45 H38 O21	914.1916	7.72	ND	2	4	3
GGG	C45 H38 O21	914.19169	6.63	1	137	319	352
GGG	C45 H38 O21	914.19184	4.37	ND	7	21	28
GGG	C45 H38 O21	914.19192	7.82	ND	1	2	1
GGGG	C60 H50 O28	1218.2508	7.33	ND	44	58	80
GGGG	C60 H50 O28	1218.25088	9.58	ND	12	5	3
GGGGG	C75 H62 O35	1522.3086	10.05	ND	2	5	8

GGGGG	C75 H62 O35	1522.30948	7.66	ND	33	52	67
GGGGGG	C90 H74 O42	1826.36757	8.90	ND	3	6	7
GGGGGGG	C105 H86 O49	2130.43022	9.17	ND	2	2	3
“G”-prodelphinidins				4	517	1112	1115
Delphinidin 3-O-(2-O-β-d-Glucopyranosyl-α-l-arabinopyranoside)	C26 H28 O16	596.13879	6.68	ND	2222	ND	ND
Anthocyanins				0	2222	0	0

Table S11. Estimated amounts (µmol/g) of major polyphenols detected in pea seed coats by the untargeted method and not quantified in Table 6. Vanillic acid 4-β-D-glucoside was used to estimate the amounts of phenolic acids, whereas kaempferol 3-O-rutinoside, quercetin 3-O-rhamnoside, myricetin 3-O-rhamnoside, luteolin 4'-O-glucoside and delphinidin 3-β-D-glucoside were used to estimate the amounts of kaempferol, quercetin and myricetin compounds, flavones and anthocyanins, respectively. Flavan-3-ols were estimated as catechin equivalents, while procyanidins, prodelphinidins and propelargonidins were estimated as procyanidin B1 equivalent.

Name	Formula	Molecular Weight	RT [min]	P1	P2	P3	P4
Dihydroxybenzoic acid	C7 H6 O4	154.02667	10.99	ND	ND	ND	6
Phenolic acid derivative	C11 H12 O5	224.06867	10.99	ND	ND	ND	ND
Phenolic acid derivative	C11 H12 O6	240.06358	8.54	ND	ND	ND	ND
Phenolic acid derivative	C12 H16 O5	240.10005	9.19	1	1	3	ND
Phenolic acid derivative	C13 H20 O4	240.13636	12.97	23	12	16	2
Phenolic acid derivative	C12 H18 O5	242.1157	5.86	12	50	12	43
Phenolic acid derivative	C12 H18 O6	258.11044	5.42	ND	1	ND	2
Phenolic acid derivative	C12 H18 O6	258.11047	6.24	ND	ND	ND	ND
Phenolic acid derivative	C12 H14 O7	270.07392	9.62	ND	ND	ND	ND
Phenolic acid derivative	C14 H24 O5	272.16243	10.59	2	ND	5	1
Phenolic acid derivative	C14 H24 O5	272.1625	9.89	13	4	16	5
Hydroxybenzoic acid hexoside	C13 H16 O8	300.08462	6.40	ND	2	ND	8
Hydroxybenzoic acid hexoside	C13 H16 O8	300.08472	3.94	23	134	15	123
Dihydroxybenzoic acid hexoside	C13 H16 O9	316.07957	8.48	5	5	6	19
Dihydroxybenzoic acid hexoside	C13 H16 O9	316.07964	8.97	1	7	ND	20
Trihydroxybenzoic acid hexoside	C13 H16 O10	332.07459	13.38	ND	3	ND	19
Phenolic acid deoxyhexoside	C15 H16 O10	356.07484	9.66	ND	ND	ND	ND
Phenolic acid derivative	C15 H18 N O9 P	387.07262	12.08	ND	2	ND	1
Hydroxybenzoic acid derivative	C16 H20 O11	388.10104	9.22	60	26	147	40

Phenolic acid derivative	C18 H28 O9	388.17373	7.18	143	496	80	301
Phenolic acid derivative	C18 H28 O10	404.16868	8.59	194	259	172	222
Phenolic acid derivative	C18 H28 O10	404.1687	11.13	37	129	51	111
5-O-[B-apiosyl-(1-2)-O-B-xylopyranosyl]gentisic acid	C17 H22 O12	418.11142	10.98	ND	ND	ND	ND
Caffeic acid malonyl glucoside	C16 H28 O13	428.15318	2.24	ND	ND	ND	ND
Hydroxybenzoic acid hexoside pentoside	C18 H24 O12	432.12679	5.74	2	1	2	2
Dihydroxybenzoic acid pentoside hexoside	C18 H24 O13	448.12215	9.43	ND	ND	ND	ND
Phenolic acid derivative	C20 H28 O14	492.14852	7.91	ND	63	ND	40
Phenolic acid derivative	C21 H32 O13	492.18471	12.43	ND	ND	6	ND
Phenolic acid dihexoside derivative	C28 H34 O18	658.17597	6.47	ND	ND	ND	ND
Phenolic acid dihexoside derivative	C29 H36 O18	672.19118	7.75	ND	ND	ND	ND
Phenolic acids				516	1195	531	965
Kaempferol acetyl hexoside	C23 H22 O12	490.11134	16.39	1	1	5	ND
Kaempferol dihexoside	C27 H30 O16	610.15424	12.47	30	16	17	34
Kaempferol malonyl hexoside	C24 H22 O14	534.10118	16.40	3	4	12	3
Kaempferol 3-O-sambioside (leucoside)	C26 H28 O15	580.1433	13.41	ND	8	ND	ND
Kaempferol pentoside-hexoside-deoxyhexoside	C32 H38 O19	726.20143	12.95	ND	ND	ND	ND
Myricetin derivative	C34 H42 O22	802.21764	11.20	ND	ND	1	ND
Myricetin 3-O-glucoside	C21 H20 O13	480.09095	12.36	ND	5	ND	7
Myricetin hexoside deoxyhexoside	C27 H30 O17	626.14864	12.19	ND	ND	ND	ND
Myricetin hexoside deoxyhexoside	C27 H30 O17	626.14869	12.11	ND	ND	ND	ND
Myricetin hexoside dideoxyhexoside	C33 H40 O21	772.20664	12.29	1365	497	12	1076
Myricetin pentoside-hexoside-deoxyhexoside	C32 H38 O21	758.19094	11.32	ND	ND	ND	ND
Myricetin pentoside-hexoside-deoxyhexoside	C32 H38 O21	758.19098	11.55	ND	ND	ND	ND
Quercetin deoxyhexoside	C21 H20 O11	448.10092	16.75	ND	ND	ND	ND

Quercetin hexoside derivative	C24 H26 O13	522.13792	10.21	ND	ND	ND	ND
Quercetin hexoside derivative	C24 H26 O13	522.13791	9.48	ND	ND	ND	ND
Quercetin pentoside	C17 H22 O13	434.10639	14.31	ND	ND	ND	ND
Quercetin pentoside-hexoside-deoxyhexoside	C32 H38 O20	742.19611	12.15	ND	ND	ND	ND
Quercetin pentoside-hexoside-deoxyhexoside	C32 H38 O20	742.19625	12.24	ND	ND	ND	ND
3,5-Dihydroxy-2-(4-hydroxyphenyl)-4-oxo-3,4-dihydro-2H-chromen-7-yl hexopyranoside	C21 H22 O11	450.11658	9.89	4	14	5	16
Flavonols				1403	545	52	1136
Tricetin hexoside	C21 H20 O12	464.09605	16.75	ND	ND	ND	ND
Tricetin hexoside	C21 H20 O12	464.09606	15.16	ND	ND	ND	2
Flavones				0	0	0	2
Afzelechin	C15 H14 O5	274.0845	10.29	ND	ND	ND	ND
Epi-afzelechin	C15 H14 O5	274.08451	11.15	ND	ND	ND	ND
(Epi)afzelechin hexoside	C21 H24 O10	436.13738	7.70	ND	ND	ND	ND
(Epi)catechin hexoside	C21 H24 O11	452.13217	6.59	ND	ND	ND	ND
(Epi)catechin hexoside	C21 H24 O11	452.13245	9.34	ND	ND	ND	ND
(Epi)gallocatechin hexoside	C21 H24 O12	468.12707	4.85	ND	ND	ND	ND
(Epi)gallocatechin hexoside	C21 H24 O12	468.12722	7.88	ND	16	ND	89
Flavan-3-ols				0	16	0	89
AA	C30 H26 O10	546.15358	11.42	ND	ND	ND	ND
AA	C30 H26 O10	546.1536	10.35	ND	ND	ND	ND
AC	C30 H26 O11	562.148	9.14	ND	ND	ND	ND
AAC	C45 H38 O16	834.2176	10.94	ND	ND	ND	ND
ACC	C45 H38 O17	850.21255	10.25	ND	ND	ND	ND
ACC	C45 H38 O17	850.21255	9.91	ND	ND	ND	ND
ACCC	C60 H50 O23	1138.27613	10.61	ND	ND	ND	ND
ACCCC	C75 H62 O29	1426.3409	11.45	ND	ND	ND	ND
Propelargonidins				0	0	0	0

CC	C30 H26 O12	578.14271	9.83	ND	ND	ND	ND
CC	C30 H26 O12	578.14298	10.40	ND	ND	ND	ND
CC	C30 H26 O12	578.14315	11.61	ND	ND	ND	ND
CCC	C45 H38 O18	866.20698	10.87	ND	ND	ND	ND
CCC	C45 H38 O18	866.207	6.03	ND	ND	ND	ND
CCC	C45 H38 O18	866.20703	9.25	ND	ND	ND	ND
CCC	C45 H38 O18	866.20715	9.73	ND	ND	ND	ND
CCCC	C60 H50 O24	1154.27068	9.84	ND	ND	ND	ND
CCCC	C60 H50 O24	1154.2707	9.97	ND	ND	ND	ND
CCCC	C60 H50 O24	1154.27127	11.69	ND	ND	ND	ND
CCCCC	C75 H62 O30	1442.33558	10.99	ND	ND	ND	ND
CCCCC	C75 H62 O30	1442.33568	10.45	ND	ND	ND	ND
CCCCCC	C90 H74 O36	1730.39828	11.68	ND	ND	ND	ND
CCCCCCC	C105 H86 O42	2018.46508	12.00	ND	ND	ND	ND
Procyanidins				0	0	0	0
GGCCC	C75 H62 O32	1474.32534	9.09	ND	ND	ND	ND
GGGGC	C75 H62 O34	1506.31495	7.97	ND	1	ND	1
GC	C30 H26 O13	594.13772	8.67	ND	ND	ND	2
GC	C30 H26 O13	594.13794	7.53	ND	2	ND	2
GC	C30 H26 O13	594.13794	6.68	ND	ND	ND	2
GC	C30 H26 O13	594.13794	7.22	ND	3	ND	5
GC	C30 H26 O13	594.13798	6.32	ND	1	ND	2
GC	C30 H26 O13	594.13803	9.01	ND	9	ND	22
GCC	C45 H38 O19	882.20148	9.54	ND	ND	ND	ND
GCC	C45 H38 O19	882.20187	8.24	ND	ND	ND	ND
GCC	C45 H38 O19	882.2021	8.36	ND	ND	ND	ND
GCC	C45 H38 O19	882.20213	5.17	ND	ND	ND	ND
GGC	C45 H38 O20	898.19605	4.78	ND	3	ND	4
GGC	C45 H38 O20	898.19622	7.43	ND	1	ND	1
GGC	C45 H38 O20	898.19641	7.34	ND	ND	ND	ND

GGC	C45 H38 O20	898.19674	9.98	ND	ND	ND	ND
GCCC	C60 H50 O25	1170.26587	9.38	ND	ND	ND	ND
GGCC	C60 H50 O26	1186.26057	9.66	ND	ND	ND	ND
GGCC	C60 H50 O26	1186.26075	8.56	ND	ND	ND	ND
GGGC	C60 H50 O27	1202.25548	7.85	ND	2	ND	1
GCCCC	C75 H62 O31	1458.33039	10.23	ND	ND	ND	ND
GGCCC	C75 H62 O32	1474.32422	10.53	ND	ND	ND	ND
GGCCC	C75 H62 O32	1474.32441	9.35	ND	ND	ND	ND
GGGCC	C75 H62 O33	1490.31944	8.78	ND	ND	ND	ND
GGGCC	C75 H62 O33	1490.31961	9.02	ND	ND	ND	ND
GGGGC	C75 H62 O34	1506.31474	8.12	ND	ND	ND	ND
GGGGC	C75 H62 O34	1506.31576	8.30	ND	ND	ND	ND
GGGCCC	C90 H74 O39	1778.38314	9.71	ND	ND	ND	ND
GGGGCC	C90 H74 O40	1794.37975	9.19	ND	ND	ND	ND
GGGGGC	C90 H74 O41	1810.37349	8.47	ND	ND	ND	ND
GGGCCCC	C105 H86 O45	2066.4502	10.55	ND	ND	ND	ND
GGGGCCC	C105 H86 O46	2082.44459	9.92	ND	ND	ND	ND
“G-C”-prodelphinidins				0	22	0	42
GG	C30 H26 O14	610.13302	6.17	ND	294	ND	314
GG	C30 H26 O14	610.13303	8.24	ND	1153	ND	1185
GG	C30 H26 O14	610.13304	4.82	ND	221	ND	200
GG	C30 H26 O14	610.13321	6.48	ND	516	ND	379
GG-deoxyhexoside	C33 H40 O20	756.21216	13.14	ND	ND	ND	ND
GGG	C45 H38 O21	914.1916	7.72	ND	493	ND	573
GGG	C45 H38 O21	914.19169	6.63	ND	466	ND	346
GGG	C45 H38 O21	914.19184	4.37	ND	289	ND	189
GGG	C45 H38 O21	914.19192	7.82	ND	24	ND	188
GGGG	C60 H50 O28	1218.2508	7.33	ND	242	ND	147
GGGG	C60 H50 O28	1218.25088	9.58	ND	220	ND	243
GGGGG	C75 H62 O35	1522.3086	10.05	ND	127	ND	216

GGGGG	C75 H62 O35	1522.30948	7.66	ND	194	ND	126
GGGGGG	C90 H74 O42	1826.36757	8.90	ND	289	ND	198
GGGGGGG	C105 H86 O49	2130.43022	9.17	ND	262	ND	131
“G”-prodelphinidins				0	4790	0	4435
Delphinidin 3-O-(2-O-β-d-Glucopyranosyl-α-l-arabinopyranoside)	C26 H28 O16	596.13879	6.68	ND	ND	ND	ND
Anthocyanins				0	0	0	0

Table S12. Estimated amounts (µmol/g) of major polyphenols detected in chickpea seed coats by the untargeted method and not quantified in Table 7. Vanillic acid 4-β-D-glucoside was used to estimate the amounts of phenolic acids, whereas kaempferol 3-O-rutinoside, quercetin 3-O-rhamnoside, myricetin 3-O-rhamnoside, luteolin 4'-O-glucoside and delphinidin 3-β-D-glucoside were used to estimate the amounts of kaempferol, quercetin and myricetin compounds, flavones and anthocyanins, respectively. Flavan-3-ols were estimated as catechin equivalents, while procyanidins, prodelphinidins and propelargonidins were estimated as procyanidin B1 equivalent.

Name	Formula	Molecular Weight	RT [min]	C1	C2	C3	C4
Dihydroxybenzoic acid	C7 H6 O4	154.02667	10.99	32	398	139	23
Phenolic acid derivative	C11 H12 O5	224.06867	10.99	ND	ND	ND	ND
Phenolic acid derivative	C11 H12 O6	240.06358	8.54	ND	ND	ND	ND
Phenolic acid derivative	C12 H16 O5	240.10005	9.19	1	ND	ND	ND
Phenolic acid derivative	C13 H20 O4	240.13636	12.97	ND	ND	ND	ND
Phenolic acid derivative	C12 H18 O5	242.1157	5.86	ND	2	2	2
Phenolic acid derivative	C12 H18 O6	258.11044	5.42	ND	ND	ND	ND
Phenolic acid derivative	C12 H18 O6	258.11047	6.24	ND	ND	ND	ND
Phenolic acid derivative	C12 H14 O7	270.07392	9.62	ND	ND	ND	ND
Phenolic acid derivative	C14 H24 O5	272.16243	10.59	3	5	8	3
Phenolic acid derivative	C14 H24 O5	272.1625	9.89	7	8	10	5
Hydroxybenzoic acid hexoside	C13 H16 O8	300.08462	6.40	ND	ND	3	ND
Hydroxybenzoic acid hexoside	C13 H16 O8	300.08472	3.94	264	13	48	25
Dihydroxybenzoic acid hexoside	C13 H16 O9	316.07957	8.48	1306	527	1836	758
Dihydroxybenzoic acid hexoside	C13 H16 O9	316.07964	8.97	170	36	455	103
Trihydroxybenzoic acid hexoside	C13 H16 O10	332.07459	13.38	70	33	672	123
Phenolic acid deoxyhexoside	C15 H16 O10	356.07484	9.66	ND	ND	ND	ND
Phenolic acid derivative	C15 H18 N O9 P	387.07262	12.08	ND	ND	ND	ND
Hydroxybenzoic acid derivative	C16 H20 O11	388.10104	9.22	109	13	12	7

Phenolic acid derivative	C18 H28 O9	388.17373	7.18	27	8	29	24
Phenolic acid derivative	C18 H28 O10	404.16868	8.59	ND	ND	ND	ND
Phenolic acid derivative	C18 H28 O10	404.1687	11.13	ND	ND	ND	ND
5-O-[B-apiosyl-(1-2)-O-B-xylopyranosyl]gentisic acid	C17 H22 O12	418.11142	10.98	ND	ND	ND	ND
Caffeic acid malonyl glucoside	C16 H28 O13	428.15318	2.24	ND	ND	ND	ND
Hydroxybenzoic acid hexoside pentoside	C18 H24 O12	432.12679	5.74	1382	244	407	329
Dihydroxybenzoic acid pentoside hexoside	C18 H24 O13	448.12215	9.43	260	77	138	96
Phenolic acid derivative	C20 H28 O14	492.14852	7.91	ND	ND	ND	ND
Phenolic acid derivative	C21 H32 O13	492.18471	12.43	90	92	74	51
Phenolic acid dihexoside derivative	C28 H34 O18	658.17597	6.47	ND	ND	ND	ND
Phenolic acid dihexoside derivative	C29 H36 O18	672.19118	7.75	ND	ND	ND	ND
Phenolic acids				3721	1456	3833	1549
Kaempferol acetyl hexoside	C23 H22 O12	490.11134	16.39	61	20	25	30
Kaempferol dihexoside	C27 H30 O16	610.15424	12.47	8	24	18	13
Kaempferol malonyl hexoside	C24 H22 O14	534.10118	16.40	148	53	63	77
Kaempferol 3-O-sambioside (leucoside)	C26 H28 O15	580.1433	13.41	1	5	5	4
Kaempferol pentoside-hexoside-deoxyhexoside	C32 H38 O19	726.20143	12.95	64	2675	3507	2586
Myricetin derivative	C34 H42 O22	802.21764	11.20	ND	1465	897	752
Myricetin 3-O-glucoside	C21 H20 O13	480.09095	12.36	ND	69	108	43
Myricetin hexoside deoxyhexoside	C27 H30 O17	626.14864	12.19	ND	1764	2639	2894
Myricetin hexoside deoxyhexoside	C27 H30 O17	626.14869	12.11	ND	3773	1725	1166
Myricetin hexoside dideoxyhexoside	C33 H40 O21	772.20664	12.29	24	7512	6787	6165
Myricetin pentoside-hexoside-deoxyhexoside	C32 H38 O21	758.19094	11.32	ND	544	967	1150
Myricetin pentoside-hexoside-deoxyhexoside	C32 H38 O21	758.19098	11.55	ND	4175	4025	3636
Quercetin deoxyhexoside	C21 H20 O11	448.10092	16.75	ND	ND	ND	ND
Quercetin hexoside derivative	C24 H26 O13	522.13792	10.21	ND	ND	ND	ND
Quercetin hexoside derivative	C24 H26 O13	522.13791	9.48	3	ND	ND	ND
Quercetin pentoside	C17 H22 O13	434.10639	14.31	ND	ND	ND	ND

Quercetin pentoside-hexoside-deoxyhexoside	C32 H38 O20	742.19611	12.15	ND	48	131	125
Quercetin-pentoside-hexoside-deoxyhexoside	C32 H38 O20	742.19625	12.24	ND	217	314	193
3,5-Dihydroxy-2-(4-hydroxyphenyl)-4-oxo-3,4-dihydro-2H-chromen-7-yl hexopyranoside	C21 H22 O11	450.11658	9.89	7	4	33	18
Flavonols				316	22348	21244	18852
Tricetin hexoside	C21 H20 O12	464.09605	16.75	ND	ND	ND	ND
Tricetin hexoside	C21 H20 O12	464.09606	15.16	ND	ND	ND	ND
Flavones				0	0	0	0
Afzelechin	C15 H14 O5	274.0845	10.29	ND	ND	ND	ND
Epi-afzelechin	C15 H14 O5	274.08451	11.15	ND	ND	ND	ND
(Epi)afzelechin hexoside	C21 H24 O10	436.13738	7.70	ND	ND	ND	ND
(Epi)catechin hexoside	C21 H24 O11	452.13217	6.59	ND	ND	ND	ND
(Epi)catechin hexoside	C21 H24 O11	452.13245	9.34	ND	ND	ND	ND
(Epi)gallocatechin hexoside	C21 H24 O12	468.12707	4.85	ND	ND	ND	ND
(Epi)gallocatechin hexoside	C21 H24 O12	468.12722	7.88	ND	4	ND	ND
Flavan-3-ols				0	4	0	0
AA	C30 H26 O10	546.15358	11.42	ND	ND	ND	ND
AA	C30 H26 O10	546.1536	10.35	ND	ND	ND	ND
AC	C30 H26 O11	562.148	9.14	ND	ND	ND	ND
AAC	C45 H38 O16	834.2176	10.94	ND	ND	ND	ND
ACC	C45 H38 O17	850.21255	10.25	ND	ND	ND	ND
ACC	C45 H38 O17	850.21255	9.91	ND	ND	ND	ND
ACCC	C60 H50 O23	1138.27613	10.61	ND	ND	ND	ND
ACCCC	C75 H62 O29	1426.3409	11.45	ND	ND	ND	ND
Propelargonidins				0	0	0	0
CC	C30 H26 O12	578.14271	9.83	ND	ND	ND	ND
CC	C30 H26 O12	578.14298	10.40	ND	ND	ND	ND
CC	C30 H26 O12	578.14315	11.61	ND	ND	ND	ND
CCC	C45 H38 O18	866.20698	10.87	ND	ND	ND	ND

CCC	C45 H38 O18	866.207	6.03	ND	ND	ND	ND
CCC	C45 H38 O18	866.20703	9.25	ND	ND	ND	ND
CCC	C45 H38 O18	866.20715	9.73	ND	ND	ND	ND
CCCC	C60 H50 O24	1154.27068	9.84	ND	ND	ND	ND
CCCC	C60 H50 O24	1154.2707	9.97	ND	ND	ND	ND
CCCC	C60 H50 O24	1154.27127	11.69	ND	ND	ND	ND
CCCCC	C75 H62 O30	1442.33558	10.99	ND	ND	ND	ND
CCCCC	C75 H62 O30	1442.33568	10.45	ND	ND	ND	ND
CCCCCC	C90 H74 O36	1730.39828	11.68	ND	ND	ND	ND
CCCCCCC	C105 H86 O42	2018.46508	12.00	ND	ND	ND	ND
Procyanidins				0	0	0	0
GGCCC	C75 H62 O32	1474.32534	9.09	ND	ND	ND	ND
GGGGC	C75 H62 O34	1506.31495	7.97	ND	ND	ND	ND
GC	C30 H26 O13	594.13772	8.67	ND	ND	ND	ND
GC	C30 H26 O13	594.13794	7.53	ND	ND	1	ND
GC	C30 H26 O13	594.13794	6.68	ND	ND	ND	ND
GC	C30 H26 O13	594.13794	7.22	ND	ND	ND	ND
GC	C30 H26 O13	594.13798	6.32	ND	ND	ND	ND
GC	C30 H26 O13	594.13803	9.01	ND	ND	ND	ND
GCC	C45 H38 O19	882.20148	9.54	ND	ND	ND	ND
GCC	C45 H38 O19	882.20187	8.24	ND	ND	ND	ND
GCC	C45 H38 O19	882.2021	8.36	ND	ND	ND	ND
GCC	C45 H38 O19	882.20213	5.17	ND	ND	ND	ND
GGC	C45 H38 O20	898.19605	4.78	ND	ND	ND	ND
GGC	C45 H38 O20	898.19622	7.43	ND	ND	ND	ND
GGC	C45 H38 O20	898.19641	7.34	ND	ND	ND	ND
GGC	C45 H38 O20	898.19674	9.98	ND	ND	ND	ND
GCCC	C60 H50 O25	1170.26587	9.38	ND	ND	ND	ND
GGCC	C60 H50 O26	1186.26057	9.66	ND	ND	ND	ND
GGCC	C60 H50 O26	1186.26075	8.56	ND	ND	ND	ND

GGGC	C60 H50 O27	1202.25548	7.85	ND	ND	ND	ND
GCCCC	C75 H62 O31	1458.33039	10.23	ND	ND	ND	ND
GGCCC	C75 H62 O32	1474.32422	10.53	ND	ND	ND	ND
GGCCC	C75 H62 O32	1474.32441	9.35	ND	ND	ND	ND
GGGCC	C75 H62 O33	1490.31944	8.78	ND	ND	ND	ND
GGGCC	C75 H62 O33	1490.31961	9.02	ND	ND	ND	ND
GGGGC	C75 H62 O34	1506.31474	8.12	ND	ND	1	ND
GGGGC	C75 H62 O34	1506.31576	8.30	ND	ND	ND	ND
GGGCCC	C90 H74 O39	1778.38314	9.71	ND	ND	ND	ND
GGGGCC	C90 H74 O40	1794.37975	9.19	ND	ND	ND	ND
GGGGGC	C90 H74 O41	1810.37349	8.47	ND	ND	ND	ND
GGGCCCC	C105 H86 O45	2066.4502	10.55	ND	ND	ND	ND
GGGGCCC	C105 H86 O46	2082.44459	9.92	ND	ND	ND	ND
“G-C”-prodelphinidins				0	0	2	0
GG	C30 H26 O14	610.13302	6.17	ND	ND	ND	ND
GG	C30 H26 O14	610.13303	8.24	ND	1	1	1
GG	C30 H26 O14	610.13304	4.82	ND	2	2	3
GG	C30 H26 O14	610.13321	6.48	ND	94	125	135
GG-deoxyhexoside	C33 H40 O20	756.21216	13.14	1	313	352	254
GGG	C45 H38 O21	914.1916	7.72	ND	ND	1	1
GGG	C45 H38 O21	914.19169	6.63	ND	79	105	110
GGG	C45 H38 O21	914.19184	4.37	ND	ND	ND	ND
GGG	C45 H38 O21	914.19192	7.82	ND	ND	ND	ND
GGGG	C60 H50 O28	1218.2508	7.33	ND	114	143	148
GGGG	C60 H50 O28	1218.25088	9.58	ND	9	13	12
GGGGG	C75 H62 O35	1522.3086	10.05	ND	15	19	12
GGGGG	C75 H62 O35	1522.30948	7.66	ND	131	168	164
GGGGGG	C90 H74 O42	1826.36757	8.90	ND	2	3	2
GGGGGGG	C105 H86 O49	2130.43022	9.17	ND	2	6	2
“G”-prodelphinidins				1	762	938	844

Delphinidin 3-O-(2-O-β-d-Glucopyranosyl-α-l-arabinopyranoside)	$C_{26}H_{28}O_{16}$	596.13879	6.68	ND	ND	ND	ND
Anthocyanins				0	0	0	0

Table S13. Estimated amounts (µmol/g) of major polyphenols detected in faba bean seed coats by the untargeted method and not quantified in Table 8. Vanillic acid 4-β-D-glucoside was used to estimate the amounts of phenolic acids, whereas kaempferol 3-O-rutinoside, quercetin 3-O-rhamnoside, myricetin 3-O-rhamnoside, luteolin 4'-O-glucoside and delphinidin 3-β-D-glucoside were used to estimate the amounts of kaempferol, quercetin and myricetin compounds, flavones and anthocyanins, respectively. Flavan-3-ols were estimated as catechin equivalents, while procyanidins, prodelphinidins and propelargonidins were estimated as procyanidin B1 equivalent.

Name	Formula	Molecular Weight	RT [min]	F1	F2	F3	F4
Dihydroxybenzoic acid	C7 H6 O4	154.02667	10.99	1	ND	3	ND
Phenolic acid derivative	C11 H12 O5	224.06867	10.99	6	354	54	130
Phenolic acid derivative	C11 H12 O6	240.06358	8.54	200	227	369	298
Phenolic acid derivative	C12 H16 O5	240.10005	9.19	72	61	293	262
Phenolic acid derivative	C13 H20 O4	240.13636	12.97	ND	ND	ND	ND
Phenolic acid derivative	C12 H18 O5	242.1157	5.86	120	147	116	138
Phenolic acid derivative	C12 H18 O6	258.11044	5.42	56	141	97	91
Phenolic acid derivative	C12 H18 O6	258.11047	6.24	620	476	392	316
Phenolic acid derivative	C12 H14 O7	270.07392	9.62	1271	1830	1437	1458
Phenolic acid derivative	C14 H24 O5	272.16243	10.59	3	5	5	2
Phenolic acid derivative	C14 H24 O5	272.1625	9.89	28	31	28	14
Hydroxybenzoic acid hexoside	C13 H16 O8	300.08462	6.40	ND	ND	6	ND
Hydroxybenzoic acid hexoside	C13 H16 O8	300.08472	3.94	8	28	74	19
Dihydroxybenzoic acid hexoside	C13 H16 O9	316.07957	8.48	14	3	43	9
Dihydroxybenzoic acid hexoside	C13 H16 O9	316.07964	8.97	2	2	12	3
Trihydroxybenzoic acid hexoside	C13 H16 O10	332.07459	13.38	ND	ND	8	2
Phenolic acid deoxyhexoside	C15 H16 O10	356.07484	9.66	ND	ND	ND	ND
Phenolic acid derivative	C15 H18 N O9 P	387.07262	12.08	141	366	438	356
Hydroxybenzoic acid derivative	C16 H20 O11	388.10104	9.22	ND	ND	ND	ND

Phenolic acid derivative	C18 H28 O9	388.17373	7.18	206	275	236	231
Phenolic acid derivative	C18 H28 O10	404.16868	8.59	44	8	41	19
Phenolic acid derivative	C18 H28 O10	404.1687	11.13	254	195	258	209
5-O-[B-apiosyl-(1-2)-O-B-xylopyranosyl]gentisic acid	C17 H22 O12	418.11142	10.98	ND	ND	ND	ND
Caffeic acid malonyl glucoside	C16 H28 O13	428.15318	2.24	68	231	321	335
Hydroxybenzoic acid hexoside pentoside	C18 H24 O12	432.12679	5.74	ND	ND	ND	ND
Dihydroxybenzoic acid pentoside hexoside	C18 H24 O13	448.12215	9.43	ND	8	156	179
Phenolic acid derivative	C20 H28 O14	492.14852	7.91	1	15	91	122
Phenolic acid derivative	C21 H32 O13	492.18471	12.43	ND	ND	ND	ND
Phenolic acid dihexoside derivative	C28 H34 O18	658.17597	6.47	ND	ND	ND	ND
Phenolic acid dihexoside derivative	C29 H36 O18	672.19118	7.75	ND	ND	ND	ND
Phenolic acids				3115	4403	4478	4193
Kaempferol acetyl hexoside	C23 H22 O12	490.11134	16.39	ND	5	ND	ND
Kaempferol dihexoside	C27 H30 O16	610.15424	12.47	402	3	2	6
Kaempferol malonyl hexoside	C24 H22 O14	534.10118	16.40	1	13	1	2
Kaempferol 3-O-sambioside (leucoside)	C26 H28 O15	580.1433	13.41	3	8	2	4
Kaempferol pentoside-hexoside-deoxyhexoside	C32 H38 O19	726.20143	12.95	4	ND	ND	ND
Myricetin derivative	C34 H42 O22	802.21764	11.20	ND	ND	ND	ND
Myricetin 3-O-glucoside	C21 H20 O13	480.09095	12.36	23	58	13	57
Myricetin hexoside deoxyhexoside	C27 H30 O17	626.14864	12.19	1	5	212	2
Myricetin hexoside deoxyhexoside	C27 H30 O17	626.14869	12.11	24	8	90	187
Myricetin hexoside dideoxyhexoside	C33 H40 O21	772.20664	12.29	1	ND	ND	ND
Myricetin pentoside-hexoside-deoxyhexoside	C32 H38 O21	758.19094	11.32	ND	ND	ND	ND
Myricetin pentoside-hexoside-deoxyhexoside	C32 H38 O21	758.19098	11.55	ND	ND	ND	ND
Quercetin deoxyhexoside	C21 H20 O11	448.10092	16.75	118	12	4	4

Quercetin hexoside derivative	C24 H26 O13	522.13792	10.21	ND	ND	ND	ND
Quercetin hexoside derivative	C24 H26 O13	522.13791	9.48	ND	ND	ND	ND
Quercetin pentoside	C17 H22 O13	434.10639	14.31	ND	ND	ND	ND
Quercetin pentoside-hexoside-deoxyhexoside	C32 H38 O20	742.19611	12.15	ND	ND	ND	ND
Quercetin pentoside-hexoside-deoxyhexoside	C32 H38 O20	742.19625	12.24	ND	ND	ND	ND
3,5-Dihydroxy-2-(4-hydroxyphenyl)-4-oxo-3,4-dihydro-2H-chromen-7-yl hexopyranoside	C21 H22 O11	450.11658	9.89	ND	1	ND	ND
Flavonols				577	113	324	262
Tricetin hexoside	C21 H20 O12	464.09605	16.75	3	ND	ND	ND
Tricetin hexoside	C21 H20 O12	464.09606	15.16	ND	9	6	9
Flavones				3	9	6	9
Afzelechin	C15 H14 O5	274.0845	10.29	ND	ND	ND	ND
Epi-afzelechin	C15 H14 O5	274.08451	11.15	ND	ND	3	2
(Epi)afzelechin hexoside	C21 H24 O10	436.13738	7.70	ND	ND	ND	ND
(Epi)catechin hexoside	C21 H24 O11	452.13217	6.59	ND	1	5	5
(Epi)catechin hexoside	C21 H24 O11	452.13245	9.34	ND	11	227	256
(Epi)gallocatechin hexoside	C21 H24 O12	468.12707	4.85	ND	ND	ND	ND
(Epi)gallocatechin hexoside	C21 H24 O12	468.12722	7.88	ND	25	220	138
Flavan-3-ols				0	37	455	401
AA	C30 H26 O10	546.15358	11.42	ND	ND	ND	ND
AA	C30 H26 O10	546.1536	10.35	ND	ND	ND	ND
AC	C30 H26 O11	562.148	9.14	ND	1	8	6
AAC	C45 H38 O16	834.2176	10.94	ND	ND	ND	ND
ACC	C45 H38 O17	850.21255	10.25	ND	ND	1	ND
ACC	C45 H38 O17	850.21255	9.91	ND	ND	1	1
ACCC	C60 H50 O23	1138.27613	10.61	ND	ND	ND	ND
ACCCC	C75 H62 O29	1426.3409	11.45	ND	ND	ND	1
Propelargonidins				0	1	10	8

CC	C30 H26 O12	578.14271	9.83	ND	31	377	269
CC	C30 H26 O12	578.14298	10.40	ND	6	47	41
CC	C30 H26 O12	578.14315	11.61	ND	8	75	66
CCC	C45 H38 O18	866.20698	10.87	ND	2	26	20
CCC	C45 H38 O18	866.207	6.03	ND	28	171	182
CCC	C45 H38 O18	866.20703	9.25	ND	21	110	119
CCC	C45 H38 O18	866.20715	9.73	ND	5	38	43
CCCC	C60 H50 O24	1154.27068	9.84	ND	ND	3	3
CCCC	C60 H50 O24	1154.2707	9.97	ND	ND	5	9
CCCC	C60 H50 O24	1154.27127	11.69	ND	11	94	89
CCCCC	C75 H62 O30	1442.33558	10.99	ND	ND	7	9
CCCCC	C75 H62 O30	1442.33568	10.45	ND	ND	7	11
CCCCCC	C90 H74 O36	1730.39828	11.68	ND	ND	8	7
CCCCCCC	C105 H86 O42	2018.46508	12.00	ND	ND	ND	1
Procyanidins				0	112	968	869
GGCCC	C75 H62 O32	1474.32534	9.09	ND	4	16	22
GGGGC	C75 H62 O34	1506.31495	7.97	ND	97	127	117
GC	C30 H26 O13	594.13772	8.67	ND	1	141	142
GC	C30 H26 O13	594.13794	7.53	ND	207	754	641
GC	C30 H26 O13	594.13794	6.68	ND	19	95	70
GC	C30 H26 O13	594.13794	7.22	ND	39	348	189
GC	C30 H26 O13	594.13798	6.32	ND	83	457	412
GC	C30 H26 O13	594.13803	9.01	ND	169	674	355
GCC	C45 H38 O19	882.20148	9.54	ND	2	11	8
GCC	C45 H38 O19	882.20187	8.24	ND	25	108	119
GCC	C45 H38 O19	882.2021	8.36	ND	71	263	276
GCC	C45 H38 O19	882.20213	5.17	ND	49	237	201
GGC	C45 H38 O20	898.19605	4.78	ND	88	228	158
GGC	C45 H38 O20	898.19622	7.43	ND	111	152	161
GGC	C45 H38 O20	898.19641	7.34	ND	19	109	52

GGC	C45 H38 O20	898.19674	9.98	ND	60	95	100
GCCC	C60 H50 O25	1170.26587	9.38	ND	5	21	20
GGCC	C60 H50 O26	1186.26057	9.66	ND	7	3	5
GGCC	C60 H50 O26	1186.26075	8.56	ND	12	20	29
GGGC	C60 H50 O27	1202.25548	7.85	ND	94	67	86
GCCCC	C75 H62 O31	1458.33039	10.23	ND	ND	6	6
GGCCC	C75 H62 O32	1474.32422	10.53	ND	3	13	8
GGCCC	C75 H62 O32	1474.32441	9.35	ND	16	44	59
GGGCC	C75 H62 O33	1490.31944	8.78	ND	1	4	2
GGGCC	C75 H62 O33	1490.31961	9.02	ND	48	111	141
GGGGC	C75 H62 O34	1506.31474	8.12	ND	97	13	13
GGGGC	C75 H62 O34	1506.31576	8.30	ND	23	7	5
GGGCCC	C90 H74 O39	1778.38314	9.71	ND	5	11	14
GGGGCC	C90 H74 O40	1794.37975	9.19	ND	10	18	20
GGGGGC	C90 H74 O41	1810.37349	8.47	ND	3	3	7
GGGCCCC	C105 H86 O45	2066.4502	10.55	ND	1	6	7
GGGGCCC	C105 H86 O46	2082.44459	9.92	ND	4	12	11
“G-C”-prodelphinidins				0	1373	4174	3456
GG	C30 H26 O14	610.13302	6.17	ND	16	82	35
GG	C30 H26 O14	610.13303	8.24	ND	182	383	176
GG	C30 H26 O14	610.13304	4.82	ND	38	139	95
GG	C30 H26 O14	610.13321	6.48	ND	110	341	233
GG-deoxyhexoside	C33 H40 O20	756.21216	13.14	ND	ND	ND	ND
GGG	C45 H38 O21	914.1916	7.72	ND	26	100	8
GGG	C45 H38 O21	914.19169	6.63	ND	97	178	127
GGG	C45 H38 O21	914.19184	4.37	ND	25	36	27
GGG	C45 H38 O21	914.19192	7.82	ND	52	100	39
GGGG	C60 H50 O28	1218.2508	7.33	ND	3	23	2
GGGG	C60 H50 O28	1218.25088	9.58	ND	7	11	5
GGGGG	C75 H62 O35	1522.3086	10.05	ND	34	4	3

GGGGG	C75 H62 O35	1522.30948	7.66	ND	11	4	5
GGGGGG	C90 H74 O42	1826.36757	8.90	ND	19	5	4
GGGGGGG	C105 H86 O49	2130.43022	9.17	ND	15	2	1
“G”-prodelphinidins				0	635	1408	760
Delphinidin 3-O-(2-O-β-d-Glucopyranosyl-α-l-arabinopyranoside)	C26 H28 O16	596.13879	6.68	ND	ND	ND	ND
Anthocyanins				0	0	0	0

Figures

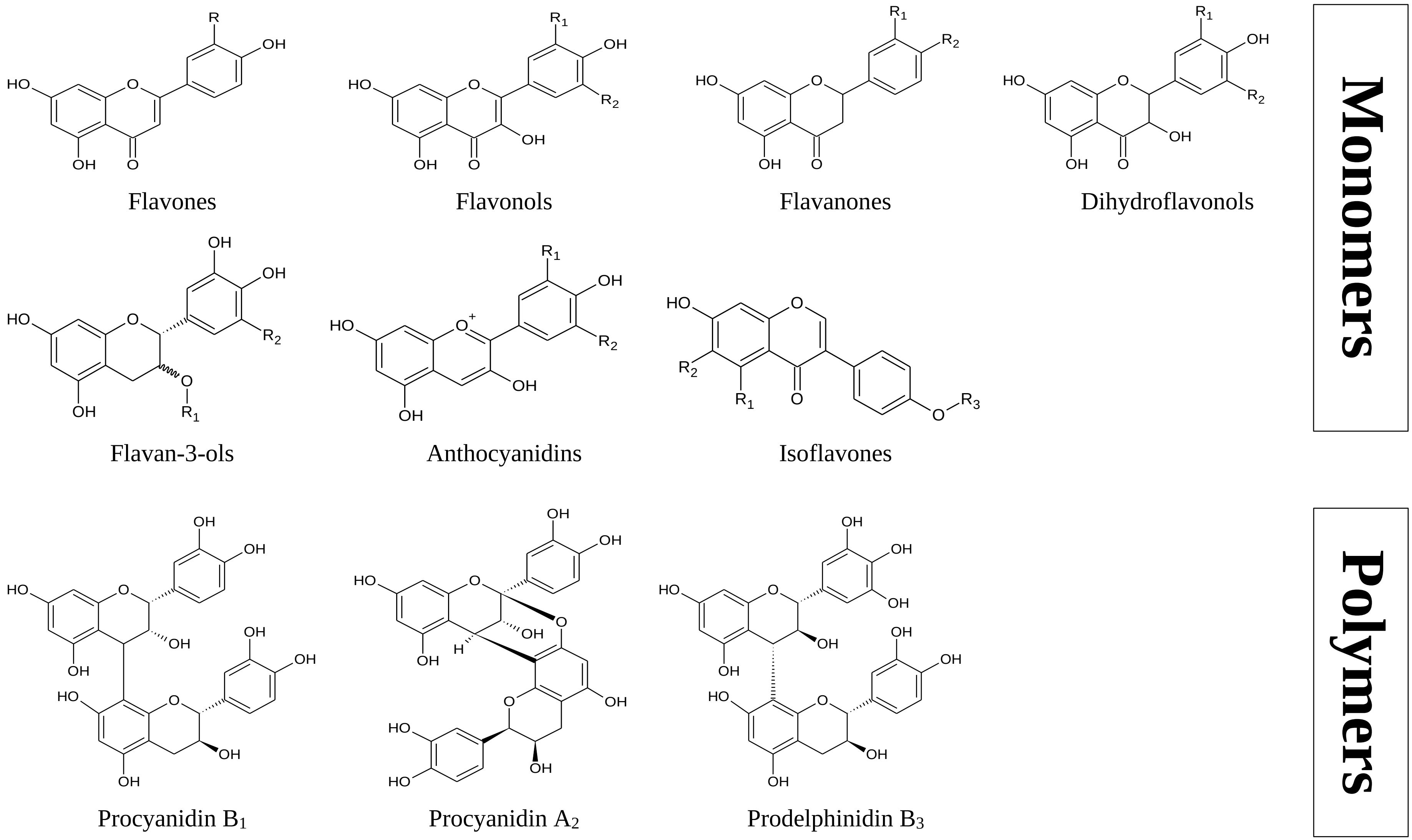

Figure S1. General structure of monomeric and examples of polymeric flavonoids

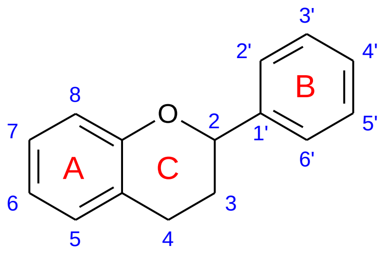

X

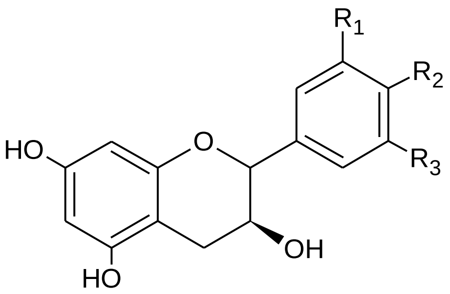

Y

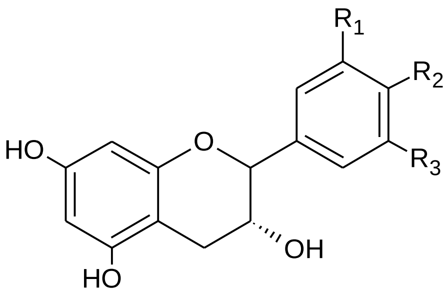

Z

	R_1	R_2	R_3
**Afzelechin**	H	OH	H
**Catechin**	OH	OH	H
**Gallocatechin**	OH	OH	OH

	R_1	R_2	R_3
**Epiafzelechin**	H	OH	H
**Epicatechin**	OH	OH	H
**Epigallocatechin**	OH	OH	OH

Figure S2. Basic structure of flavonoid rings (X), and flavan-3-ol monomers (Y and Z) that polymerize to form different types of proanthocyanidin polymers

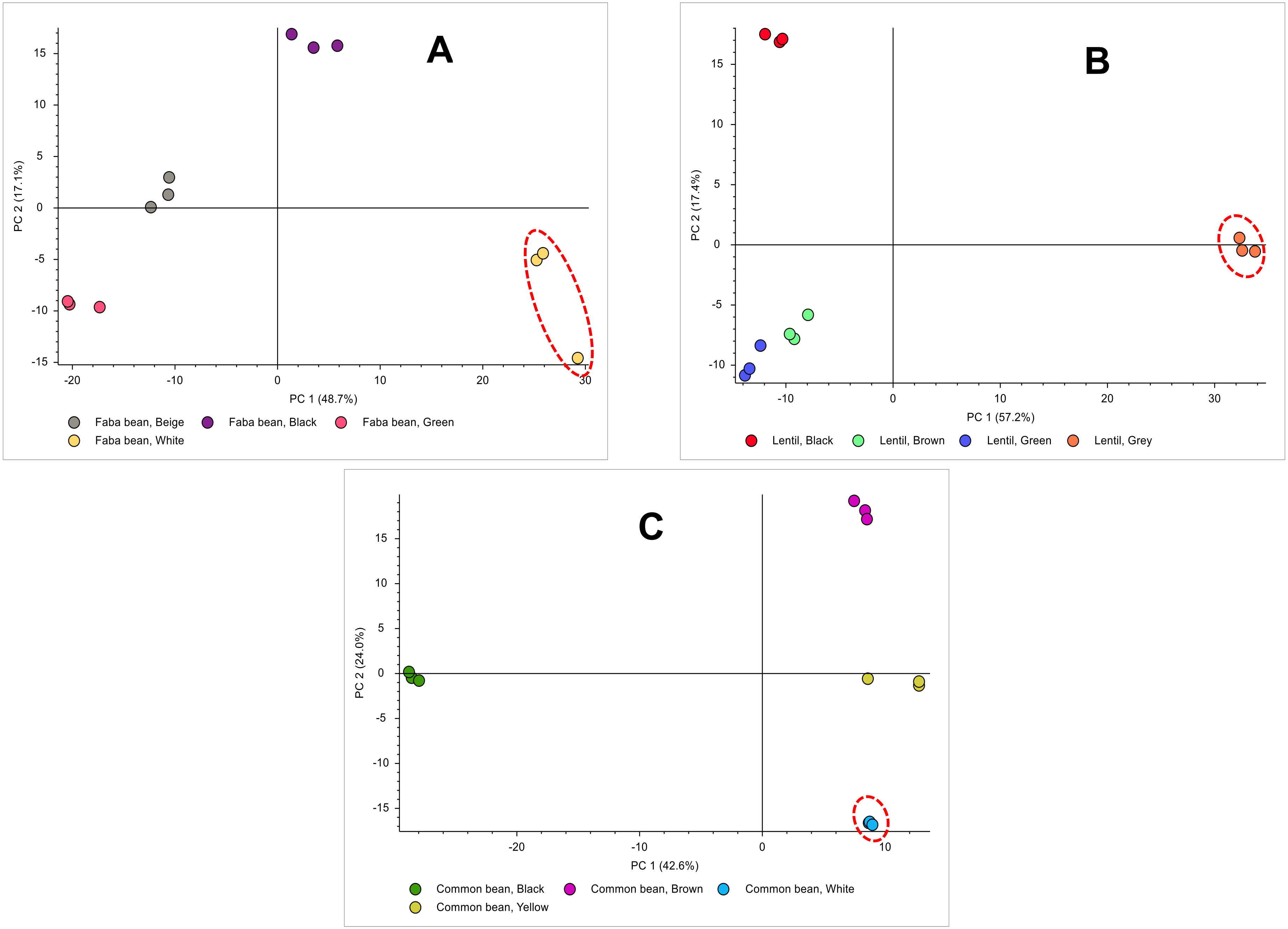

Figure S3. A Principal component analysis (PCA) plot of PC1 versus PC2 of faba bean **(A)**, lentil **(B)** and common bean **(C)** seed coats. Dotted red circles refer to the low tannin genotypes.

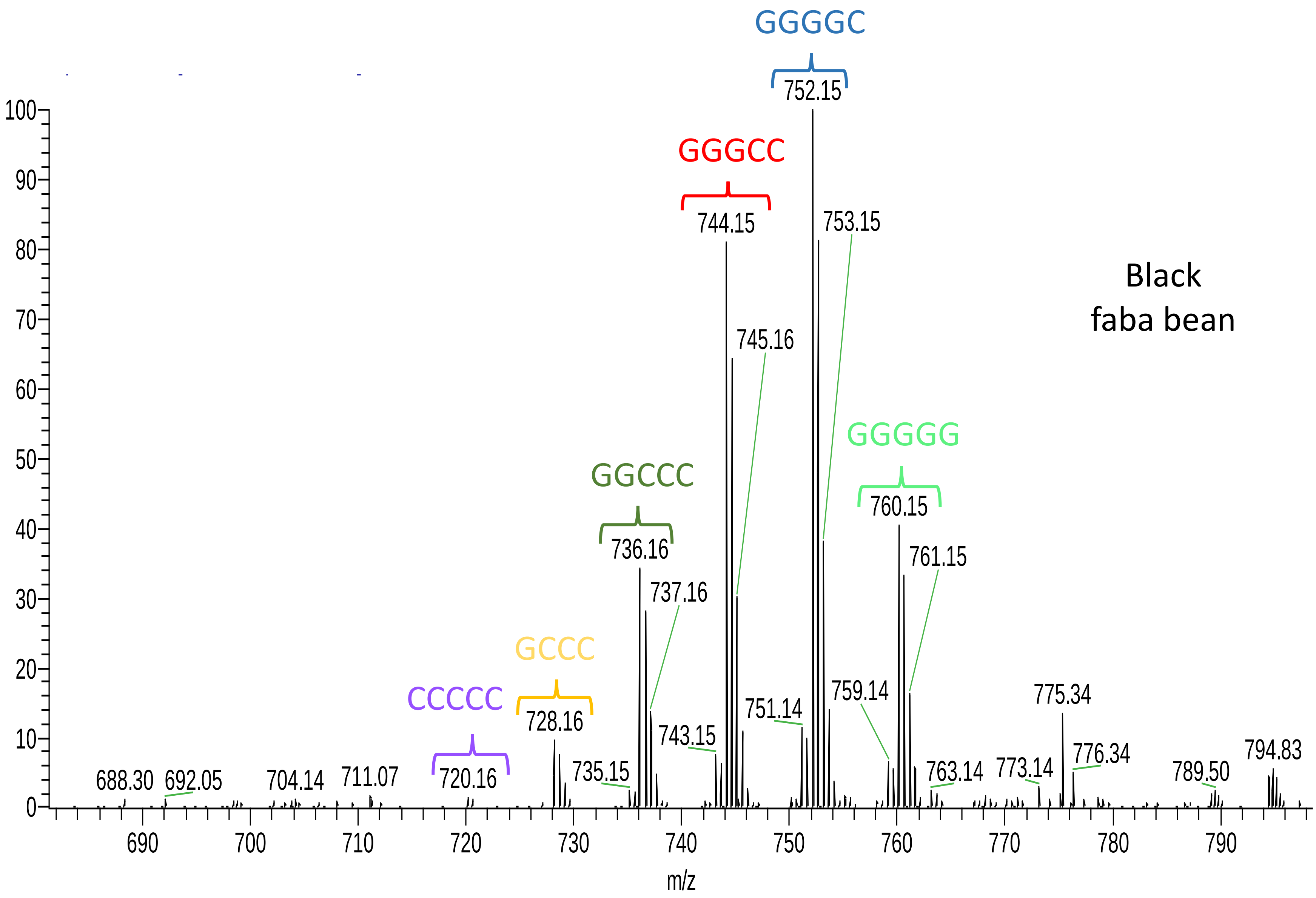

Figure S4. HRMS full scan showing a procyanin pentamer and different prodelphinidin pentamers detected in faba bean seed coats
